# Accelerated production of human epithelial organoids in a miniaturized spinning bioreactor

**DOI:** 10.1016/j.crmeth.2024.100903

**Published:** 2024-11-18

**Authors:** Shicheng Ye, Ary Marsee, Gilles S. van Tienderen, Mohammad Rezaeimoghaddam, Hafsah Sheikh, Roos-Anne Samsom, Eelco J.P. de Koning, Sabine Fuchs, Monique M.A. Verstegen, Luc J.W. van der Laan, Frans van de Vosse, Jos Malda, Keita Ito, Bart Spee, Kerstin Schneeberger

**Affiliations:** 1 Department of Clinical Sciences, Faculty of Veterinary Medicine, Utrecht University, Uppsalalaan 8, Utrecht 3584 CT, the Netherlands; 2 Department of Biomedical Engineering, Eindhoven University of Technology, Eindhoven 5600 MB, the Netherlands; 3 Department of Internal Medicine, Leiden University Medical Center, P.O. Box 9600, Leiden 2300 RC, the Netherlands; 4 Hubrecht Institute, KNAW (Royal Netherlands Academy of Arts and Sciences), Utrecht 3584 CT, the Netherlands; 5 Department of Metabolic Diseases, Wilhelmina Children’s Hospital, University Medical Center Utrecht, Lundlaan 6, Utrecht 3584 EA, the Netherlands; 6 Department of Surgery, Erasmus MC Transplant Institute, University Medical Center Rotterdam, P.O. Box 2040, Rotterdam 3000 CA, the Netherlands; 7 Department of Orthopedics, University Medical Center Utrecht, Utrecht University, Utrecht 3584 CX, the Netherlands

**Keywords:** organoid, bioreactor, suspension culture, RPMotion, stem cell

## Abstract

Conventional static culture of organoids necessitates weekly manual passaging and results in nonhomogeneous exposure of organoids to nutrients, oxygen, and toxic metabolites. Here, we developed a miniaturized spinning bioreactor, RPMotion, specifically optimized for accelerated and cost-effective culture of epithelial organoids under homogeneous conditions. We established tissue-specific RPMotion settings and standard operating protocols for the expansion of human epithelial organoids derived from the liver, intestine, and pancreas. All organoid types proliferated faster in the bioreactor (5.2-fold, 3-fold, and 4-fold, respectively) compared to static culture while keeping their organ-specific phenotypes. We confirmed that the bioreactor is suitable for organoid establishment directly from biopsies and for long-term expansion of liver organoids. Furthermore, we showed that after accelerated expansion, liver organoids can be differentiated into hepatocyte-like cells in the RPMotion bioreactor. In conclusion, this miniaturized bioreactor enables work-, time-, and cost-efficient organoid culture, holding great promise for organoid-based fundamental and translational research and development.

## Highlights

• We develop a miniaturized spinning bioreactor for human epithelial organoid culture
• The bioreactor can ensure homogeneous fluidic distribution and uniform flow
• The method is time, labor, and cost efficient
• Applicable to a wide variety of organoids, holding great promise for organoid-based R&D

## Motivation

Organoids are widely utilized for disease modeling, drug selection, and developmental studies and are promising for regenerative therapies. Expanding organoids in a conventional three-dimensional (3D) culture, using basement membrane extract hydrogels (e.g., Matrigel) in culture plates, is labor intensive and costly. Commercially available spinner flask-based methods are suitable for large-scale (billions of cells) production of organoids, but the associated high costs can hamper translational studies. To overcome these limitations, we developed a miniaturized spinning bioreactor for the effective production of organoids in suspension, reducing time, labor, and costs.

## Graphical abstract

**Figure:**
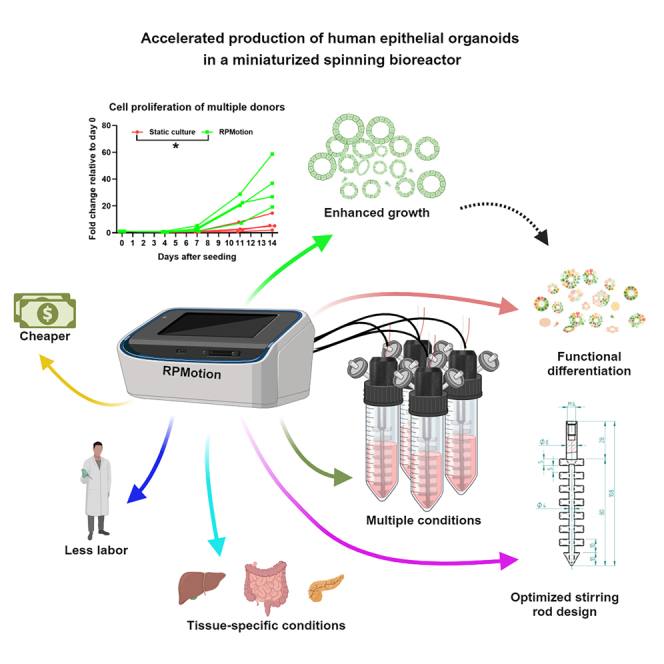

## Teaser

Ye et al. develop a miniature spinning bioreactor, RPMotion, to efficiently expand human organoids of different tissue origin, including liver, pancreas, and intestine. The RPMotion can be employed to rapidly and cost-efficiently produce human epithelial organoids for a broad range of applications.

## Introduction

Organoids can be established from primary differentiated cells and from two main stem cells of various species: adult stem cells^1^^,^^2^^,^^3^^,^^4^^,^^5^^,^^6^^,^^7^ and pluripotent stem cells (PSCs), including embryonic stem cells and induced PSCs.^8^^,^^9^^,^^10^ Of note, adult stem cell-derived epithelial organoids can be expanded for several months, biobanked, and genetically manipulated,^11^^,^^12^ which facilitates the study of human biology and gene editing for regenerative medicine purposes. As such, organoids are valuable as *in vitro* models for fundamental and translational research and development (R&D), and as cell sources for future transplantation purposes.^12^^,^^13^^,^^14^^,^^15^^,^^16^^,^^17^

To generate sufficient cell numbers for these diverse applications, efficient production of adult stem cell-derived organoids (e.g., epithelial organoids) becomes increasingly critical. However, conventional methods-based extensive expansion of organoids is an expensive and tedious process, requiring not only monotonous labor time, but also expensive resources such as medium components and basement membrane hydrogels (e.g., Matrigel and equivalent). The process involves embedding cells in a static droplet of Matrigel submerged in media containing growth factors and other important molecules.^1^^,^^2^^,^^4^^,^^11^ This static culture method necessitates weekly passaging and results in the localized buildup of toxic metabolites, as well as nonhomogeneous nutrient and oxygen distribution. Importantly, weekly passaging can be a shortcoming hampering clinically relevant production times for regenerative medicine applications.^18^ Bioreactors are advantageous over static droplet-based cultures by increasing the transport of oxygen and nutrition and minimizing gradient formation (e.g., pH, metabolites, and dissolved oxygen).^19^^,^^20^ Therefore, various large-scale bioreactors, including spinning bioreactors, have been used for (stem) cell culture.^21^^,^^22^^,^^23^^,^^24^^,^^25^ For example, spinning bioreactors were shown to be efficient for the generation of kidney and brain organoids,^26^^,^^27^^,^^28^^,^^29^ and a spinner flask-based method was established for large-scale production of intrahepatic cholangiocyte organoids (ICOs).^18^ Although those methods are of great value for future clinical applications where billions of cells are needed, the yield generally exceeds the need for fundamental and translational R&D applications. Another drawback of using the currently available large-scale bioreactors is their large inoculation volume, which hampers testing different culture conditions, including different rotational speeds and starting cell concentrations. Thus, there is a critical need to develop a miniaturized bioreactor that is suitable for the accelerated production of multiple organoid types.

Here, we engineer a miniaturized spinning bioreactor, RPMotion, using three-dimensional (3D) printing technologies. The RPMotion bioreactor is specifically developed for organoid suspension culture in 50-mL vessels. We first optimize protocols for the expansion and differentiation of ICOs. Then, we expand the application of the bioreactor for the rapid production of human organoids derived from the small intestine and pancreas. The bioreactor enables rapid and cost-effective production of multiple types of organoids, indicating broad applications in regenerative medicine, particularly in fundamental and translational R&D.

## Results

### Setup of the RPMotion bioreactor and rotor selection

**Figure 1:**
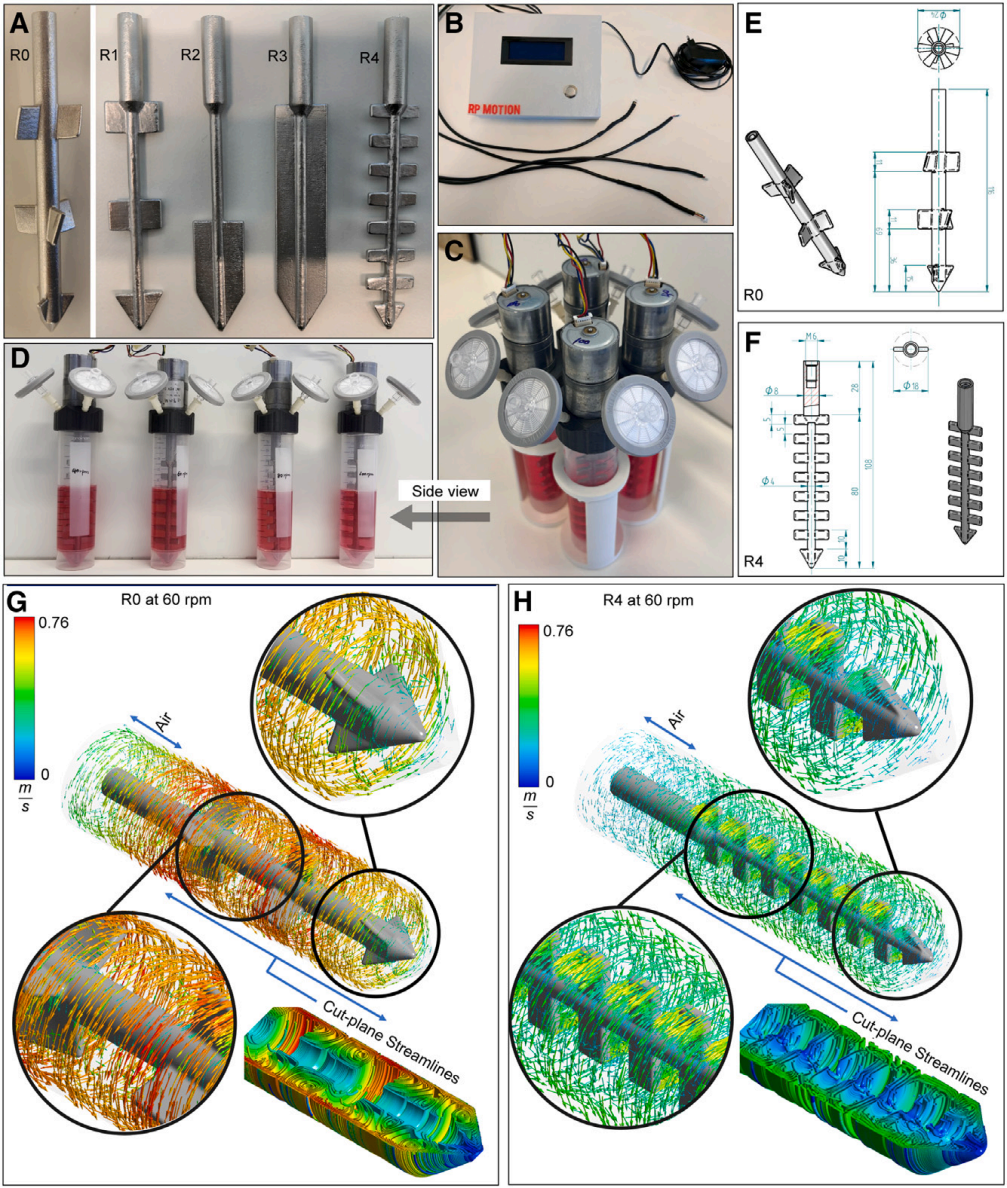
Design of the RPMotion bioreactor and computational fluid dynamics analysis (A) Five designs of rotors (R0, R1, R2, R3, R4) were tested for the RPMotion bioreactor, and R4 was eventually chosen for further experiments. (B) The control enclosure was placed outside of the incubator, with wires connecting to the bioreactors inside the incubator. The hardware enclosure contains an Arduino Uno, an LCD screen, a push-button, a 12-V power source, motor connections, and associated components. (C and D) Top (C) and (D) side view of one assembled RPMotion unit. Each unit can run up to four bioreactors, which sit in a custom 3D-printed holder. One bioreactor consists of one 50-mL Falcon tube, one rotor connected to one motor, which was controlled by an individual wire from the control unit, and two 0.22-μm filters on the side of the lid to allow for gas exchange. (E and F) Detailed designs of R0 and R4. Parameters include the lengths of the rotors, the widths of the rotors and their blades, the numbers of blades, and the distance between blades. (G and H) Vector field visualization for R0 and R4. Computational results, rotor tip, and middle closed views are also shown as well as the cut plane velocity streamlines.

The RPMotion system consists of two main parts: (1) four stirred bioreactors, which contain cells in suspension inside a standard incubator, and (2) a control unit outside the incubator, which is used to control the operation of the bioreactors. One bioreactor is made up of a standard 50-mL Falcon vessel, two standard 0.22-μm filters integrated into the custom-printed lids for gas exchange, and a stainless-steel rotor (R) (Figure 1A) connected to the external control unit (Figure 1B) containing an Arduino Uno microcontroller board and a liquid-crystal display (LCD) screen. Each control unit of the RPMotion system can run up to four bioreactors that sit in a custom 3D-printed holder (Figures 1C and 1D). The motors, push-button, LCD screen, and Arduino Uno are connected to one another in a circuit (Figure S1C). The software for running different rotational programs of the RPMotion bioreactor is shown in Figure S2.

The RPMotion rotors are tailored for organoid culture. For the evaluation of different rotor designs we used ICOs. To select the best rotor for organoid culture, we first compared five rotor designs (R0–4 shown in Figure 1A) for organoid culture. We found that there was no obvious difference between tilted (R0) vs. flat (R1) propeller blades in regard to cell yields (Figure S3A and S3B). However, the number and spacing of the propeller blades influenced the homogeneous distribution of organoids, as well as cell proliferation in the RPMotion, with the rotor design with more blades (R4) resulting in less organoid aggregation and increased numbers of cells (Figures S3C and S3D). To further explore the factors influencing organoid aggregation and expansion, we employed computational fluid dynamics (CFD) analysis to compare parameters including fluid velocity and shear strain between the original rotor R0 (Figure 1E) and the rotor yielding the highest cell numbers, R4 (Figure 1F). In order to validate our computational model with experimental data, we compared the volume fraction of the medium-air mixture and the shapes of the free surface for both rotor designs with the RPMotion running at 60 rpm, while utilizing the same 40-mL volume of the medium. The chosen medium volume corresponds to a height of 94 mm within the Falcon vessel for R0 and 89 mm for R4 due to differences in rotor volumes. The free-surface shape results of the CFD modeling exhibited a qualitative agreement with the experimental data for both R0 and R4 (Figures S1D and S1E). Analysis of the flow field, which includes velocity vectors, path lines, and fluid strain rate, revealed that with R0, a higher-magnitude velocity field was created, reaching a maximum of 0.76 m/s (Figures 1G and 1H). With R4, the fluid velocity distribution was predicted to be more homogeneous, with lower average and maximum velocity magnitudes. Furthermore, examining the path lines on a cut plane, R4 created a symmetric flow pattern along with uniform secondary flow and recirculation zones. Conversely, R0 generated stronger vortices due to the angle of attack and slightly twisted blades, resulting in a non-homogeneous mixing pattern. Additionally, the triple-tapered blades at the tip of the rotor caused a downward flow and created a dead spot with less re-circulation at the bottom of the falcon tube. This phenomenon could have contributed to more unwanted cell clustering with R0. The magnitude of the strain rates was not different between rotors, but R4 created larger volumes of higher strain rates and a more well-mixed environment throughout the entire vessel (Figures S1F and S1G). In summary, the mechanical characteristics and the modeled homogeneous flow pattern in R4 were in line with our experimental data that showed a homogeneous distribution of organoids in R4 and a higher yield of organoids compared to the other rotors. Therefore, we utilized R4 for further experiments. In single-donor experiments, we determined optimal rotational speeds for efficient ICO expansion (60 rpm) and differentiation (100 rpm), human small intestinal organoid (HSIO) expansion (100 rpm) and differentiation (80 rpm), and human pancreatic ductal organoid (PDO) expansion (40–100 rpm) (data not shown).

### ICOs expand 5.2-fold faster in the RPMotion bioreactor compared to static culture

**Figure 2:**
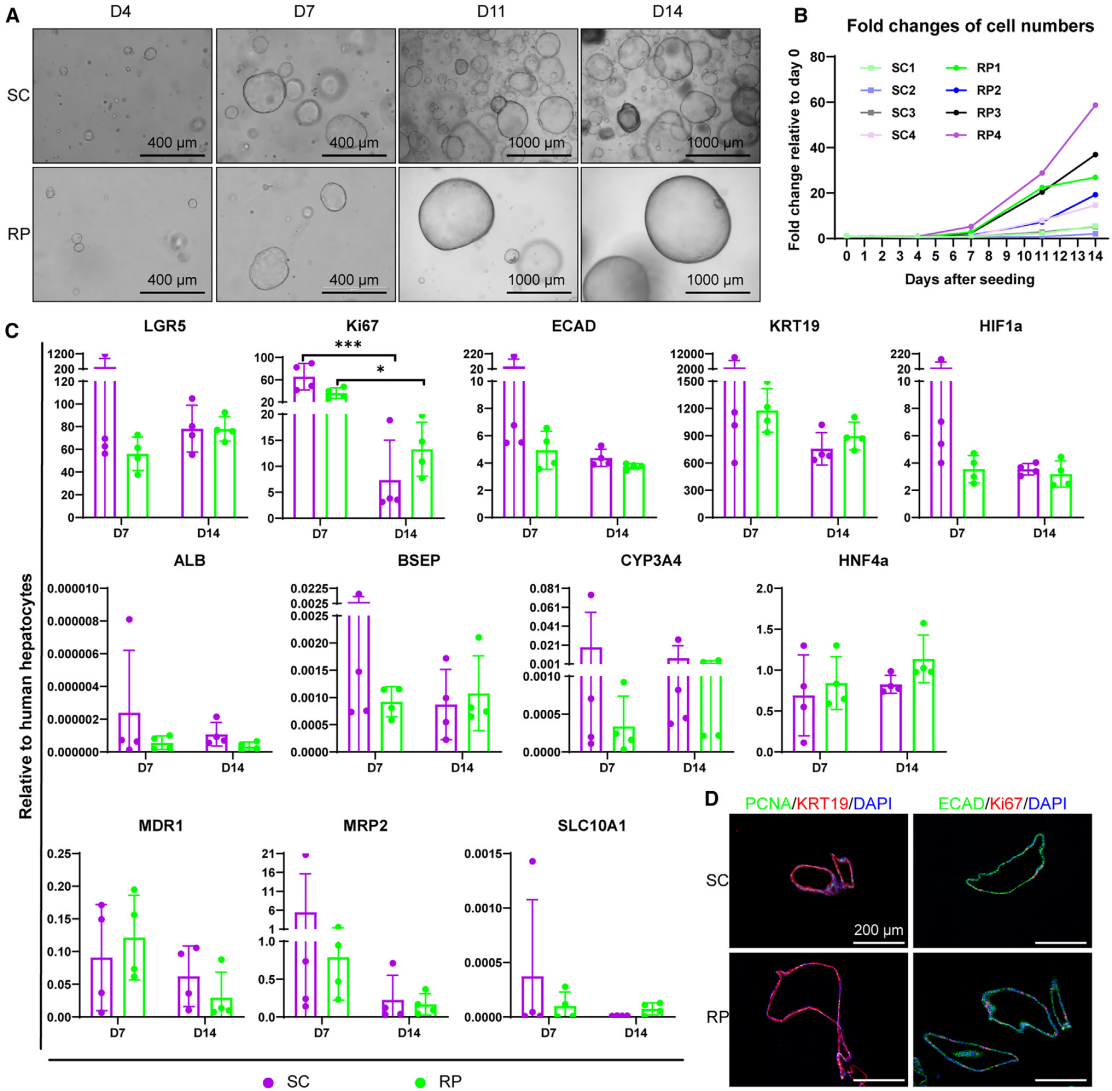
ICOs expand faster in the RPMotion bioreactor (A) Morphology of ICOs expanded in static culture (SC) and in the bioreactor (RP) at 60 rpm. Bright-field images were taken on D4, D7, D11, and D14 after single-cell seeding. Scale bars, 400 μm (D4 and D7) and 1,000 μm (D11 and D14). (B) Fold changes of cell proliferation in static culture and in the bioreactor at 60 rpm. Four donors were used (with numbers 1, 2, 3, and 4). (C) Gene expression of ICOs cultured in static culture and in the bioreactor with expansion medium. Šídák’s multiple comparisons test was applied. Graphs indicate mean ± SD. ∗*p* < 0.05; ∗∗∗*p* < 0.0005. (D) Characterization of ductal (KRT19), epithelial (ECAD), and proliferative (Ki67 and PCNA) markers of ICOs expanded in expansion medium by immunofluorescent (IF) staining. Scale bar, 200 μm.

With R4 selected for organoid culture, we validated with four different ICO lines in the bioreactor at 60 rpm and compared it to static culture. Bright-field images showed that the diameters of ICOs were comparable in all conditions on day (D) 4 and D7, while on D11 and D14, ICOs expanded in bioreactors reached larger diameters than those in static culture (Figure 2A). For quantification, we conducted cell-counting assays after dissociation of organoids at four time points (D4, D7, D11, and D14). Compared to the conventional static culture method, we observed an average 5.2-fold faster organoid expansion in the bioreactor (Figure 2B). Gene expression profiling (Figure 2C) and immunofluorescent (IF) staining assays (Figure 2D) were used to characterize the ICOs in static and dynamic conditions. The qPCR results showed that ICOs under dynamic conditions maintained the expression of stem/progenitor cell marker leucine-rich repeat containing G protein-coupled receptor 5 (*LGR5*) during 2 weeks of culture at similar levels as static conditions, while the expression of the proliferative marker (marker of proliferation Ki-67, *Ki67*) was downregulated on D14 compared to D7 in both dynamic and static conditions (Figure 2C). The epithelial (cadherin 1, *ECAD*), cholangiocyte (keratin 19, *KRT19*), hypoxia (*HIF1A*), and hepatic (hepatocyte nuclear factor 4 alpha, *HNF4A*) markers were all comparably expressed in ICOs expanded in static culture and in the bioreactor (Figure 2C). Compared to the gene expression of human hepatocytes, ICOs expanded in the bioreactor and the static culture retained very low expression of differentiation markers, such as albumin (*ALB*), ATP binding cassette subfamily B member 11 (*ABCB11*, also known as *BSEP*), cytochrome P450 family 3 subfamily A member 4 (*CYP3A4*), mitochondrial 37S ribosomal protein MRP2 (*MRP2*), ATP binding cassette subfamily B member 1 (*ABCB1*, also known as *MDR1*), and solute carrier family 10 member 1 (*SLC10A1*) (Figure 2C). IF images showed that ductal (KRT19), epithelial (ECAD), and proliferative (proliferating cell nuclear antigen [PCNA] and Ki67) proteins were detected in ICOs expanded in both static culture and the bioreactor (Figure 2D). These results confirmed that ICOs expanded in the bioreactor maintained their epithelial, ductular, and proliferative phenotypes.

### ICOs can be differentiated into hepatocyte-like cells in the RPMotion bioreactor

**Figure 3:**
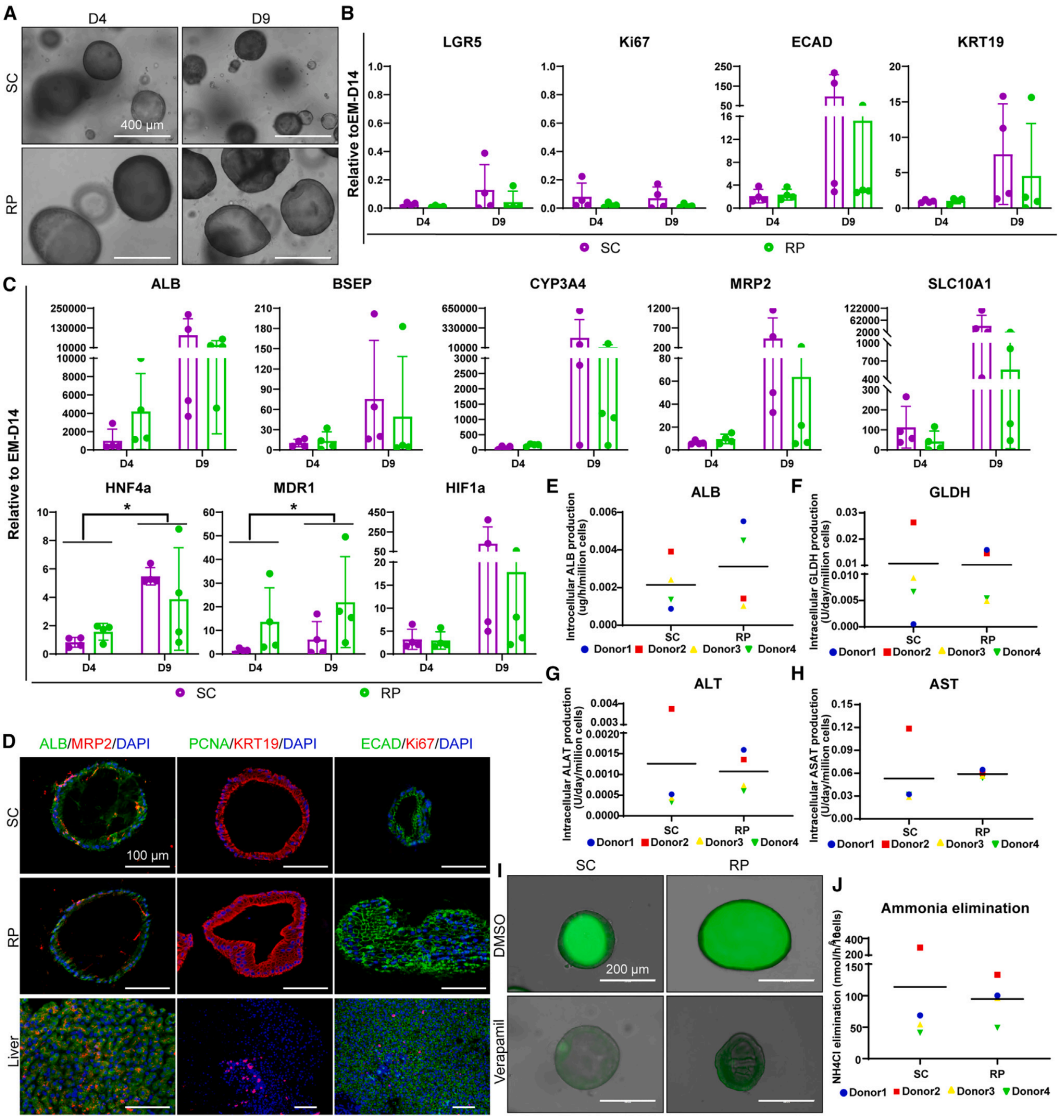
ICOs can be differentiated into hepatocyte-like cells in the RPMotion bioreactor (A) Morphology of ICOs differentiated in static culture and in the bioreactor at 100 rpm. Bright-field images were taken at D4 and D9 of differentiation. Scale bar, 400 μm. (B and C) Gene expression of ICOs cultured in static culture and in the bioreactor with differentiation medium. Šídák’s multiple comparisons test was applied. Graphs indicate mean ± SD. ∗*p* < 0.05. (D) Characterization of ECAD, KRT19, and hepatic (ALB and MRP2) and proliferative (Ki67 and PCNA) markers in differentiated organoids by IF staining. Scale bar, 100 μm. (E–H) Intracellular levels of (D) albumin, (E) GLDH, (F) ALT, and (G) AST of differentiated organoids. (I) Rhodamine 123 transport assay to assess the function (transporter protein, MDR1) of differentiated ICOs. Scale bar, 200 μm. (J) Ammonia elimination of differentiated ICOs; *n* = 4.

We further validated the bioreactor for differentiation of four ICO lines toward hepatocyte-like cells at 80 rpm. After 4 days of differentiation, ICOs became condensed and looked darker under the microscope, and this morphological change continued until the end time point D9 in both the bioreactor and the static culture, with larger-diameter ICOs in the bioreactor (Figure 3A). Bright-field images were not indicative enough to confirm the differentiation of ICOs toward hepatocyte-like cells. Thus, we conducted gene expression analysis with qPCR assays. Compared to the gene expression of ICOs in the expansion medium (EM) at D14, both stem/progenitor cell marker *LGR5* and proliferative marker *Ki67* were downregulated, while the epithelial marker *ECAD* and ductal marker *KRT19* were upregulated after differentiation (Figure 3B). All hepatic markers including *ALB*, *BSEP*, *CYP3A4*, *MRP2*, and *SLC10A1* were much higher expressed than in EM conditions and showed a trend of upregulation between D4 and D9, and, of these, *HNF4a* and *MDR1* were significantly upregulated (Figure 3C). The hypoxia marker *HIF1a* was also upregulated after differentiation, possibly due to the denser morphology of the organoids. The overall gene expression of ICOs differentiated in the bioreactor and the static culture were comparable. We then characterized the differentiated ICOs with IF staining assays. IF images confirmed the presence of hepatic proteins (ALB^+^, MRP2^+^), the absence of proliferative proteins (PCNA^−^, Ki67^−^), and the maintenance of a ductal (KRT19^+^), epithelial (ECAD^+^) phenotype (Figure 3D). Furthermore, we quantified the intracellular levels of major hepatocyte proteins based on their protein concentration, such as ALB, glutamate dehydrogenase (GLDH), alanine transaminase (ALT), and aspartate transaminase (AST) (Figures 3E–3H). The results showed that the levels of all these proteins were similar in ICOs differentiated in static culture and in the bioreactor (Figures 3E–3H). Moreover, we conducted Rhodamine 123 transport assays to determine functional multidrug resistance 1 (MDR1)-dependent transmembrane transport. First, we pretreated ICOs with either verapamil, a competitive inhibitor of MDR1, or DMSO as a control. Then, we incubated ICOs with the medium containing Rhodamine 123, a fluorescent compound that can be exported by MDR1 to the apical lumen of the ICOs. The representative images showed that Rhodamine 123 was accumulated in the lumen of ICOs pretreated with DMSO, while ICOs pretreated with verapamil retained Rhodamine 123 in the cells, indicating that the transport was MDR1 specific (Figure 3I). Furthermore, we assessed the ammonium elimination capacity of ICOs after differentiation and found that ICOs differentiated in both static culture and the bioreactor showed comparable ammonium elimination, suggesting functional maturation of differentiated ICOs (Figure 3J). All the results above confirmed the suitability of using the RPMotion bioreactor for the differentiation of ICOs toward hepatocyte-like cells.

### The RPMotion bioreactor is suitable for long-term expansion of ICOs

To confirm the feasibility of long-term culture of organoids in the bioreactor, we expanded four ICO lines in both the bioreactor and the static culture for four rounds (8 weeks), with passaging after each round of expansion. Although there were some differences in regard to the diameters of organoids by the end (D14) of each round of expansion, the overall diameters of organoids in the bioreactor were larger than in static culture (Figure S4A). In addition to the morphological pictures, we quantified the fold change of cell numbers during each round of organoid expansion and found that organoid proliferation was consistent over four rounds of expansion (Figure S4B). Of note, to verify that the bioreactor is suitable for organoid expansion from single cells as well as from fragments, we mechanically dissociated the organoids into fragments at the end of round 2 and started round 3 of organoid culture with organoid fragments in both the bioreactor and the static culture (Figure S4B). The proliferative curves indicated that for organoids started from fragments, the fold changes of cell numbers increased earlier than starting from single cells. This can possibly be explained with a faster recovery from the passaging stress of fragments compared to single cells. The overall proliferation rate was similar to that started from single cells, and the final fold changes of cell numbers were comparable (between 3- and 4.2-fold) in all rounds of organoid expansion (Figure S4C). However, we also found that the donor-to-donor variations were larger than starting with single cells, which could be caused by a less-homogeneous starting population of organoid fragments. Accumulatively, the bioreactor resulted in a more than 200 times higher cell mass than the static culture after 8 weeks of organoid expansion (Figure S4D). Next, we characterized the gene expression of organoids collected over four rounds of expansion with qPCR and IF stainings. qPCR results showed that the expression levels of most genes tested were comparable between the bioreactor and static culture and were stable over four rounds of expansion (Figure S4E). Interestingly, some genes (*LGR5*, *KRT19*, *HIF1a*, and *ALB*) were differentially expressed between certain rounds of static culture, while no significant differences were observed in the RPMotion bioreactor (Figure S4E). IF images showed that the presence of epithelial (ECAD), ductal (K19), and proliferative (Ki67 and PCNA) proteins are all similar between organoids expanded in the bioreactor and the static culture in different rounds of expansion (Figure S4F). Therefore, the bioreactor is suitable for organoid culture from single cells or fragments and is efficient for long-term expansion of organoids.

### Establishment of ICO lines from human liver biopsies in the bioreactor

We further tested the feasibility of establishing new ICO lines directly from biopsies in the bioreactor. We utilized human liver biopsies from four donors (one fresh tissue from donor no. 1085 and three frozen tissues from donors nos. 1395, 12, 13) and started with the same number of tissue samples in both the bioreactor and the static culture. After 7 days of culture, we saw organoids formed from three donors (nos. 1085, 1395, 13) in the static culture and two donors (nos. 1085 and 1395) in the bioreactors (Figure S5A). On D14, only the tissue from donor 12 had not resulted in organoid formation, either in the bioreactor or the static culture. The other three donors generated organoids, with organoids in the bioreactor showing larger diameters (Figure S5B). To compare the efficiency of organoid establishment in the bioreactor with that in the static culture, we collected all organoids from the bioreactors and the static cultures on D14 (passage 0; p0), passaged them, and reseeded them as fragments in new Matrigel droplets for semi-quantitative analysis. The bright-field images taken at D4 after passaging showed that all three ICO lines had formed cystic organoids in passage 1 (p1) (Figure S5C). Based on the morphological pictures, there was a trend of more organoids formed from fragments derived from the bioreactor conditions compared to the static conditions. The overall morphology of organoids derived from the bioreactor and the static culture were similar (Figure S5C).

### Efficient expansion and differentiation of HSIOs in the bioreactor

To probe the potential of the RPMotion bioreactor to be used for organoids of other organs/tissues, we proceeded to culture human small intestinal organoids (HSIOs) and human PDOs. For HSIO expansion, we inoculated the bioreactors with the same starting cell concentration as for ICOs, using human small intestinal organoid EM and 100 rpm as the rotational speed. Similar to ICOs, HSIOs reached comparable diameters in the bioreactor and static conditions by D7, whereafter HSIOs in the bioreactor showed a sharp increase in diameters. Different from ICOs, HSIOs in the static culture became so confluent that they adapted a thick and dark morphology, and we had to passage them on D12 (Figure S6A). In contrast, HSIO cultures in the bioreactor could be continued until D14 (data not shown). Cell-counting results showed that the average fold changes of cell numbers compared to D0 reached around 15 and 45 by D12 in the static culture and the bioreactor, respectively (Figure S6B). To further characterize the organoids, we analyzed gene expression levels of stem/progenitor cell markers (*AXIN2*, *LGR5*), the proliferative marker *Ki67*, the epithelial marker e-cadherin (*ECAD*), the robust stem cell marker olfactomedin 4 (*OLFM4*), and the goblet cell marker mucin 2 (*MUC2*). qPCR results showed that compared to gene expression of the static culture on D7, expression levels were comparable between D7 and D14 and mostly comparable between static culture and the bioreactor (Figure S6C). Only *AXIN2* was lower expressed in the bioreactor than in the static culture on D14, and *OLFM4* was higher expressed in the bioreactor than in the static culture on D7 (Figure S6C).

By the end of the expansion on D14, we changed the EM to differentiation medium supplemented with 10% (v/v) Matrigel and continued HSIO differentiation for 7 days, using a rotational speed of 80 rpm in the bioreactor. Samples were collected for RNA isolation on D3, D5, and end time point D7. Interestingly, HSIOs seemed to keep growing until D3 of differentiation (DM-D3) without much change in their morphology (Figure S6D). Starting from D5, HSIOs showed a darker and thicker morphology, with some budding structures. This morphological change was even more pronounced on D7 (Figure S6D). Strikingly, compared to gene expression on DM-D0, the stem cell markers *AXIN2*, *LGR5*, and *OLFM4* and proliferative marker *Ki67* were increased at DM-D3, before they continuously decreased at DM-D5 and DM-D7, as expected (Figure S6E). However, the epithelial marker *ECAD*, the goblet cell marker mucin 2 (*MUC2*), and hypoxia marker *HIF1a* showed a trend of upregulation between D3 and D7 (Figure S6E). HSIOs in the bioreactor displayed higher expression levels of *ECAD* and microvillus marker villin 1 (*VIL1*), but also hypoxia marker *HIF1a* compared to static culture (Figure S6E). To further characterize the differentiated HSIOs on DM-D5 and DM-D7, we conducted IF staining assays. IF images showed that HSIOs differentiated in both the bioreactor and the static culture expressed the epithelial marker ECAD, the Paneth cell marker lysozyme (LYZ), and the microvillus protein VIL1 at DM-D5 and DM-D7 (Figures S6F and S6G). Few Ki67^+^ cells could be detected in the static condition at DM-D5, whereas Ki67 protein was absent in the static condition at DM-D7 and in the bioreactor at both time points, DM-D5 and DM-D7 (Figure S6G). Thus, the bioreactor can be applied for efficient HSIO expansion and for HSIO differentiation.

### Accelerated expansion of PDOs in the bioreactor

Next, we expanded PDO lines from four different donors in the bioreactor. Since PDOs are sensitive to single-cell passaging, we started the cultures with small fragments, using a CytoSmart cell counter for quantification. Light microscopy pictures showed that on D4, PDOs in static culture were larger than in the bioreactor (Figure S7A). On D7, the diameters in both conditions seemed comparable, and after D7, PDOs cultured in the bioreactor showed a sharp increase in organoid diameters (Figure S7A) and cell numbers (Figure S7B), as previously observed for ICOs and HSIOs. Although PDO diameters were large and heterogeneous in both conditions due to passaging as fragments, the overall diameters of PDOs in the bioreactor were larger than in the static culture. This large organoid diameter might also have caused the observed plateau in cell numbers in the static culture between D11 and D14, while PDOs in the bioreactor kept expanding until D14 (Figure S7B). With donor no. 143 considered an outlier and removed from the cell number calculations, the final fold changes of cell numbers in the static culture and in the bioreactors were 5.39 and 21.9, respectively. We then conducted qPCR assays for *LGR5*, *Ki67*, SRY-box transcription factor 9 (*SOX9*), *ECAD*, pancreatic and duodenal homeobox 1 (*PDX1*), insulin (*INS*), and *HIF1a* to characterize the PDOs produced in both conditions. Expression levels of all genes were comparable between D7 and D14, and almost all the genes showed similar expression levels in static culture and the RPMotion, except for *KRT19* and *ECAD*, which were lower in the bioreactor than in static culture on D7. Furthermore, we characterized the PDOs with IF staining assays. The IF images confirmed the presence of proliferative marker proteins Ki67 and PCNA, epithelial marker protein ECAD, and ductal markers K19 and SOX9, the latter of which is also a pancreatic progenitor marker. These results suggest that the bioreactor is also suitable for efficient PDO expansion.

## Discussion

In this study, we successfully developed a miniaturized spinning bioreactor, RPMotion, which enables the accelerated production of human organoids derived from the liver, pancreas, and intestine. On average, ICOs, HSIOs, and PDOs expanded in the RPMotion displayed approximately 5.2-, 3-, and 4-fold faster proliferation, respectively, compared to static controls (Figures 2B, S6B, and S7B). The faster expansion of organoids in the spinning bioreactor is in line with our previous study with large-scale spinner flasks.^18^ Although the exact underlying mechanisms contributing to the increased expansion in the bioreactor remain to be demonstrated, we expect that improved dissolved oxygen and homogeneous distribution of soluble factors are contributing factors.^19^^,^^20^ These possible factors can be characterized with advanced sensor technologies in the future.^23^^,^^30^^,^^31^^,^^32^ Other reports confirm the advantages of using suspension bioreactors for organoid culture over static cultures. For example, spinning bioreactors were shown to be efficient for the generation of kidney and brain organoids,^25^^,^^28^^,^^33^^,^^34^ bioreactors were suitable for long-term maintenance of retinal organoids and can improve photoreceptor yields,^35^^,^^36^ vertical-wheel bioreactors were utilized for scalable production of PSCs and their differentiation into different types of organoids,^37^ and rotating wall bioreactors accelerated the growth and maturation of PSC-derived intestinal organoids.^38^ However, all those bioreactors are either not efficient for organoid expansion or associated with large volumes of cell suspension, making their use expensive.

The RPMotion bioreactor bridges a gap between bioreactors of micro-scale with a volume ranging from less than 1 mL to a few milliliters and large-scale bioreactors with a volume from 125 mL to 2 L. Previous publications introduced the application of micro-scale spinning bioreactors in modeling development and disease with cerebral organoids^39^ or to generate human brain region-specific organoids.^27^ These bioreactors are suitable for the long-term maintenance of organoids, but they are not suited to produce organoids on a larger scale due to the small volumes (200–500 μL). Moreover, according to Qian et al., they are “not currently available for mass production” and “the assembly procedures are tedious.”^27^ Some large spinning bioreactors can produce organoids on a relatively large scale^18^^,^^33^; however, the yield and associated costs are far too high for organoid applications in R&D. The bioreactor developed in this study utilizes standard 50-mL Falcon vessels, which is convenient and sufficient for most applications in R&D. For instance, with an average yield of 15–20 million cells per bioreactor after 2 weeks of culture, the RPMotion can be used for biobanking and (personalized) drug testing or gene editing. A summary comparing the RPMotion bioreactor with other methods for organoid culture is shown in Figure S8.

The successful culture of organoids derived from the liver, pancreas, and intestine indicates broad applications of the bioreactor for multiple downstream applications, particularly for efficient biobanking. Currently reported spinning bioreactors for organoid culture are mostly validated for only one type of organoid, and the organoids are derived mostly from (induced) PSCs.^27^^,^^33^^,^^36^^,^^38^^,^^39^^,^^40^ To the best of our knowledge, the RPMotion bioreactor is the first reported spinning bioreactor that enables faster growth and efficient differentiation of organoids derived from more than three different human tissues, including the liver, intestine, pancreas, and kidney (unpublished data). We also show the feasibility of long-term expansion of ICOs in the bioreactor and the possibility of starting organoid culture from organoid fragments (Figure S4). Moreover, we successfully established new organoid lines from fresh and frozen human liver tissues directly in the bioreactor (Figure S5), confirming that the RPMotion can fully replace the conventional static culture method. The fold changes of cell numbers show that multiple donor lines of organoids derived from different tissues expanded in the bioreactors grew in similar patterns compared to those in the static cultures, with fast-growing lines resulting in higher cell numbers both in the bioreactor and in static cultures and slow-growing lines resulting in fewer cells, respectively. This indicates that the donor-specific variation in proliferation rate is retained under these dynamic conditions, although overall cellular yield is always higher compared to static conditions. In addition, we found that the variations in fold changes of cell numbers are larger among different donors (Figures 2B, S6B, and S7B) than at different speeds (data not shown), indicating that a range of rotational speeds is suitable for the culture of various cystic organoids in the bioreactor. Overall, different organoids grew well at a speed of 40–100 rpm, particularly around 60–80 rpm. A lower speed may not be enough for the later stage of expansion, while a too high speed could cause too much stress at the start of the culture, when the organoid formation could be affected. Therefore, in the future, it would be interesting to increase the speed gradually during the culture period.

In the protocol presented here, culture medium and Matrigel was added every 2–3 days without the need to remove medium from the culture. Depending on study-specific needs, researchers can also choose to remove a certain amount of supernatant before adding fresh medium and Matrigel to the organoid suspension to reduce the amount of waste products in the medium. Furthermore, depending on the growth rate and associated organoid density in the suspension, organoids in the bioreactor can be passaged at an earlier or later time point than the time frame of 2 weeks that we have presented. Currently, animal-derived matrices (e.g., Matrigel and equivalent) are still the hydrogels that are mostly used for organoid culture^16^^,^^41^; these are not suitable for clinical applications due to their ill-defined composition and large batch-to-batch variations. Therefore, it will be necessary to replace animal-derived hydrogels with synthetic hydrogels or human-derived materials for organoid culture in the bioreactor in the future.^42^^,^^43^^,^^44^ Well-defined synthetic hydrogels for organoid cultures can not only avoid ethical issues, including the animal-derived and ill-defined shortcomings, but also enable the study of specific cell-extracellular matrix interactions, and pave the way for organoids to be utilized in therapeutic applications. Furthermore, the RPMotion bioreactor can also be used as a model to optimize protocols for larger bioreactors for organoid culture. For example, to adjust the organoid culture process to be good manufacturing practice compliant for clinical applications.^45^ Organoids tested in this study were derived from healthy tissue and typically grow as single-layer structures with cystic lumens. In the future, it will be interesting to test tumoroids or organoids derived from other pathological tissues in the RPMotion bioreactor and to establish protocols for organoids with solid morphology.

In conclusion, we demonstrated the suitability of using the RPMotion bioreactor to culture different types of human organoids. As the bioreactor enables (long-term) faster expansion and efficient differentiation of organoids compared to the conventional static culture, we believe that the ease of use and scalability of the RPMotion bioreactor offer unprecedented opportunities such as efficient biobanking and a broad range of downstream applications of organoids in R&D.

### Limitations of the study

Although the RPMotion bioreactor enables faster expansion of ICOs, HSIOs, and PDOs compared to static cultures, the underlying mechanisms for the faster proliferation remain to be elucidated. Moreover, we did not observe better differentiation of organoids as did previous reports about other spinning bioreactors (e.g., to induce maturation of retinal organoids).^35^^,^^36^ Also, as it is not required to remove media from the bioreactor during culture, it may be beneficial for organoids to maintain their specific microenvironment. However, this may also result in the accumulation of waste produced by cells, which needs to be overcome in the future. Furthermore, this study was limited to the expansion of cystic growing organoids. It will be interesting to apply the bioreactor to other organoid types in the future, including organoids with solid morphology and diseased and (induced) PSC-derived organoids.

## Resource availability

### Lead contact

Requests for protocols and reagents can be directed to Kerstin Schneeberger (k.schneeberger@uu.nl).

### Materials availability

No unique reagents were generated in this study. All materials used in the study were resourced from commercial vendors, except for the 3D-printed materials, homemade media, and organoids, which were derived from healthy tissues of patients upon consent. Details about the materials can be provided upon request by the lead contact, Kerstin Schneeberger (k.schneeberger@uu.nl).

### Data and code availability

• All data generated in this study are available within the article or from the lead contact upon request.
• This study reports a combination of existing and original code to run the RPMotion. The code is deposited at https://zenodo.org/records/13938310.
• Any additional information needed to reanalyze the data reported in this paper is available from the lead contact by request.

## Acknowledgments

We thank I. F. Schene for providing human small intestinal organoids, J. Juksar for providing human pancreatic organoids, and N.D.A. Nieuwenhuijze for sharing the Wnt-conditioned medium. We thank R.S. Schwanen (10.13039/501100001829Utrecht University) and Dr. Dongli Liang (Instrumental Analysis Center, 10.13039/501100004921Shanghai Jiao Tong University) for their help with the revision of the manuscript. The authors further acknowledge the partnership with 10.13039/100019247Ansys. This work was supported by the Dutch Research Council NWO VENI (016.Veni.198.021) to K.S. and the 10.13039/501100004543China Scholarship Council (CSC201808310180) to S.Y. The graphical abstract and Figure S8 were created with BioRender.com.

## Author contributions

S.Y., G.S.V.T., B.S., and K.S. conceptualized the RPMotion system and method. S.Y. and A.M. optimized the method. S.Y. designed and performed all the cell-related experiments. K.I. and M.R. designed the CFD analysis, and M.R. analyzed the CFD data. H.S., R.-A.S., and F.v.d.V. carried out the experiments and data collection. S.Y. performed the data analyses and wrote the manuscript, and M.R. contributed to the draft of the CFD part. E.J.P.d.K. provided the PDOs. S.F. provided the HSIOs. M.M.A.V. and L.J.W.v.d.L. provided the human liver biopsies for ICOs generation. K.S. and B.S. supervised this study. All authors provided critical feedback and helped shape the research and manuscript.

## Declaration of interests

S.Y., G.S.v.T., B.S., and K.S. hold a Dutch patent, NL2029095B1, and a PCT patent application, WO2023031329A1, on the organoid bioreactor with royalties paid. K.S. and B.S. are founders, employees, and shareholders of the Utrecht University spin-off company Orgonex B.V.

## STAR★Methods

### Key resources table

**Table:** 

REAGENT or RESOURCE	SOURCE	IDENTIFIER
**Antibodies**		

Anti-Ki67	Thermo Scientific	RM-9106-S; RRID:AB_2341197
Anti-E-cadherin	BD Bioscience	610181; RRID:AB_397580
Anti-PCNA	Santa Cruz	sc-56; RRID:AB_628110
Anti-K19	Abcam	ab76539; RRID:AB_1523469
Anti-ALB	Sigma	A6684; RRID:AB_258309
Anti-MRP2	Abcam	ab187644; RRID:AB_3665246
Anti-LYZ	Abcam	ab74666; RRID:AB_1310422
Anti-VIL-1	Abcam	ab201989; RRID:AB_3665247
Anti-SOX9	LSbioscciences	LS-C148618; RRID:AB_11130372
Anti-mouse Alexa 488	ThermoFisher	A-11029; RRID:AB_2534088
Anti-rabbit Alexa 488	ThermoFisher	A-11034; RRID:AB_2576217
Anti-mouse Alexa 568	ThermoFisher	A-11004; RRID:AB_2534072
Anti-rabbit Alexa 568	ThermoFisher	A-11036; RRID:AB_10563566

**Chemicals, peptides, and recombinant proteins**		

HepatiCult™ Organoid Kit (Human)	Stem Cell Technologies	Catalog # 100-0386
Human HepatiCult™ Organoid Growth supplement	Stem Cell Technologies	Catalog # 100-0389
HepatiCult™ Organoid Differentiation supplement	Stem Cell Technologies	Catalog # 100-0388
Human IntestiCult™ Organoid Growth medium	Stem Cell Technologies	Catalog # 06010
Human IntestiCult™ Differentiation Medium	Stem Cell Technologies	Catalog # 100-0214
Basal medium (Advanced DMEM/F12 (AD))	Thermo Fisher Scientific	Catalog # 12634028
GlutaMax	Gibco	Catalog # 35050061
HEPES	Gibco	Catalog # 15630-056
Penicillin-streptomycin	Gibco	Catalog # 15140-122
N2	Fisher Scientific	Catalog # 17-502-001
B27	Thermo Scientific	Catalog # 17504044
N-Acetylcysteine	Sigma-Aldrich	Catalog # A7250
RSPO1 conditioned media	Home made	NA
Wnt3a conditioned media	Home made	NA
Recombinant human (Leu15)-gastrin I	Tebu-Bio	Catalog # AS-64149
EGF	Invitrogen	Catalog # PHG0313
Noggin	Peprotech	Catalog # 120-10C
fibroblast growth factor 10 (FGF10)	Peprotech	Catalog # 100-26
Nicotinamide	Sigma-Aldrich	Catalog #N0636-100
A83.01	Stem Cell Technologies	Catalog # 72022
Forskolin (FSK)	Sigma-Aldrich	Catalog #F3917-25MG
Prostglandin E2 (PGE2)	R&D Systems	Catalog # 2296/10
Y27632 dihydrochloride	Selleck Chemicals	Catalog #S1049
Gefitinib	Axon Med chem	Catalog # Axon 1393
GS inhibitor XX (GSI-XX)	Sigma-Aldrich	SML0649-5MG
Ethanol 70%	Any supplier	NA
Matrigel	Corning	Catalog # 356231
Trypan Blue solution 0.4%	Gibco	Catalog # 15250061
CTS TrypLE Express Enzyme no phenol red	Fisher Scientific	Catalog # 12604013
Anti-adherence rinsing solution	Stemcell Technologies	Catalog # 07010

**Biological samples**		

Human liver tissues	Provided by the Erasmus Medical Center Rotterdam	NA

**Experimental models: cell lines**		

Human small intestinal organoid (HSIO) lines	Provided by the group of prof. Sabine Fuchs	NA
Human pancreatic ductal organoid (PDO) lines	Provided by the group of prof. Eelco J. P. de Koning	NA

**Oligonucleotides**		

Primers for qPCR assays are listed in Table 2	This paper	N/A

**Software and algorithms**		

ImageJ	https://imagej.net/	ImageJ 1.51j8
Prism 9	GraphPad	N/A
Excel	Microsoft	N/A
Ansys SpaceClaim	ANSYS, Inc	SpaceClaim 2023R1
Ansys Fluent	ANSYS, Inc	Fluent 2023R1
Arduino script	This paper	Zenodo: https://zenodo.org/records/13938310

**Other**		

BioRad Cell counter	BioRad	TC 20™ Automated Cell Counter
CytoSmart Cell counter	CytoSmart	CytoSmart Exact FL

### Experimental model and study participant details

#### Human intrahepatic cholangiocyte organoid (ICO) establishment

Human liver biopsies were obtained from donor liver during liver transplantation procedures at the Erasmus Medical Center Rotterdam. Use of the biopsies for research purposes is in accordance with the ethical standard of the Helsinki Declaration of 1975. The use of the tissue for research purposes was approved by the Medical Ethical Council of the Erasmus Medical Center and by the liver transplant recipient (MEC-2014-060). Donor age and sex were not considered in this study. ICO lines were established as described.^4^ In short, liver biopsies were cut into small pieces, followed by the enzymatic digestion with type II collagenase (0.125 mg/mL, Gibco) and dispase (0.125 mg/mL, Gibco) in DMEM GlutaMAX (Gibco) containing 1% (v/v) fetal calf serum (FCS, Gibco). The supernatant was collected every hour. Tissue digestion followed by supernatant collection was performed three times. Collected single cells were washed in DMEM GlutaMAX (Gibco) containing 1% (v/v) FCS (Gibco) and centrifuged for 5 min at 400 g. The cells were resuspended in Matrigel (Corning) at a concentration of ∼500 cells/μL. Cells were seeded in droplets (50 μL) in non-attaching 24-well plates (M9312, Greiner, Merck). EM was added after approximately 15 min incubation at 37°C, 5% CO_2_ in air. For experiments displayed in Figure S5, (ICO establishment in the bioreactor), small pieces of liver biopsies were digested with Tryple-Expression for 15–20 min at 37°C, and then both single cells and residual biopsies were collected and directly used for organoid formation in the RPMotion or static conditions as described.

#### Organoid expansion and differentiation

For ICO expansion and differentiation, HepatiCult Organoid Kit (Human) (Stem Cell Technologies, Catalog # 100–0386) was used.

To compare ICO expansion in the RPMotion and static culture, single cells were prepared from organoids by trypsinizing with TrypLE Express Enzyme (12604-013, Gibco). For RP, 5x10^5^ cells were added to commercial Human HepatiCult Organoid Growth Medium (Expansion medium, EM), containing 10% (v/v) Matrigel. The initial total volume for RP was 5 mL. For static culture, single cells were seeded in droplets (50 μL/well) at a concentration of 1,000 cells/μL, in temperature and humidity-balanced 24-well plates (M9312, Greiner, Merck). After seeding, plates were incubated at 37°C for 15-30 min to facilitate Matrigel gelation, and thereafter, 500 μL EM was added to each well. For the first three days of culture, 10 μM of Y-27632 (SelleckChem) was added to the medium. EM was refreshed every 2–3 days. All cultures were incubated at 37°C, 5% CO_2_ in air.

For ICO differentiation, the medium was changed, after 14 days of expansion, from commercial EM to HepatiCult Organoid Differentiation Medium (Differentiation medium, DM) for both static culture and RPMotion. DM was refreshed every 2 days for 8–9 days.

Timelines of medium addition and removal are listed in the table below.

**Table:** Timeline of medium addition and removal

Day	Removal (mL) + outputs	Addition of medium, mL	Addition of Matrigel, mL	Total, mL
0	–	4.5 EM (with cells)	0.5	5
2	–	2.7 EM	0.3	8
4	0.5 for cell counting	4.05 EM	0.45	12
7	0.5 for cell counting and 1.5 for RNA isolation	5.4 EM	0.6	16
9	–	8.64 EM	0.96	25.6
11	0.5 for cell counting	13.5 EM	1.5	40.1
14	0.5 for cell counting + 1.5 for RNA isolation + 2 for fixation and stainings	the residual organoids can be collected by centrifugation, then filtering through a 70-μm cell strainer (not necessary if a clearly compact pellet after centrifugation was shown) to start differentiation		
0	change to DM on D14 of EM	9 DM	1	10
2	–	2.7 DM	0.3	13
4	1 for fixation and stainings + 2 for RNA isolation	2.7 DM	0.3	13
6	–	3.6 DM	0.4	17
8	All	sample collection or preparation for functional assays		

For human small intestine organoid (HSIO) culture, Human IntestiCult Organoid Growth medium (Expansion medium, EM, Catalog # 06010) and Differentiation Medium (DM, Catalog # 100–0214) were applied. The starting and collection of HSIO culture were similar to ICO culture. Briefly, 5x10^4^ cells/well were seeded for static culture, and 5x10^5^ cells/tube were started in 5 mL of medium containing 10% (v/v) Matrigel for RPMotion. Morphological pictures were taken after 4, 7, and 12 days of single-cell seeding and cell counting was conducted after taking photos, respectively. HSIOs in the static culture were passaged on D12 of expansion to avoid over confluency. Similarly, after 14 days of expansion, EM was changed to DM for starting differentiation. DM was refreshed every 2 days for 7 days.

For pancreatic ductal organoids (PDOs) culture, EM composition for PDO expansion was prepared as previously described^46^: Basal medium (Advanced DMEM/F12 (AD, Gibco), containing 1% (v/v) GlutaMax (Gibco), HEPES (10 mM, Gibco) and 1% (v/v) penicillin-streptomycin (Gibco, Dublin, Ireland)) supplemented with 1X N2 (Gibco) and 1X B27 (Gibco), 1.25 mM N-Acetylcysteine (Sigma-Aldrich), 10% (v/v) RSPO1 conditioned media (homemade), 10 nM recombinant human (Leu15)-gastrin I (GAS, Sigma-Aldrich), 50 ng/mL EGF (Peprotech), 25 ng/mL Noggin (Peprotech), 100 ng/mL fibroblast growth factor 10 (FGF10, Peprotech), 10 mM Nicotinamide (Sigma-Aldrich), 5 μM A83.01 (Tocris), 10 μM Forskolin (FSK,Tocris) and 3 μM Prostglandin E2 (PGE2, Tocris)]. 10 μM Rho Kinase inhibitor (Y27632, Sigma-Aldrich) was added for the first 7 days. The starting and collection of PDO culture were also similar to ICO culture. Due to the sensitivity of PDOs to enzyme treatment, fragments instead of single cells were used for cell counting and initiation in both RPMotion and static culture. The other difference was 3x10^4^ cells/well were seeded for static culture and 3x10^5^ cells/tube were started in 5 mL of medium containing 10% (v/v) Matrigel for RPMotion. Other procedures were the same as HSIO culture.

### Method details

#### Materials and assembly of the miniature spinning bioreactor, RPMotion

Detailed information on the materials and the assembly of the RPMotion (RP) was shown in Figure S1 (Figure S1). Parts needed to assemble the RPMotion (excluding enclosure and motor connections) were depicted in Figure 1A. Top row from left to right shows: 3D-printed custom holder fitting 4 bioreactors; Standard 50mL flat-bottom conical vessel; 3D printed rotor; 12V DC brushless motor connected to 3D printed bioreactor lid; male-to-female leurers; 0.22 μM sterile filters. Middle row from left to right: 4 screws; 10k Ω resistor; Jumper wires; Screw terminals; Push-button; LCD screen. Bottom row from left to right: USB to USB connector; 5V USB adaptor plug; 12V AC/DC converter; Arduino Uno; 400 tie-point interlocking solderless breadboard. The motors, pushbutton, LCD screen and the Arduino Uno are connected to each other in circuit (Figure S1C). The control box is placed outside the incubator and connected with power as well as motors inside the incubator. The software to run the RPMotion is included as Figure S2.

#### Computational fluid dynamics (CFD) analysis

CFD analysis was conducted to analyze the flow characteristics that could potentially influence organoid aggregation in RPMotions with two different rotors, R0 and R4. First, CAD files for both rotors were imported into Ansys SpaceClaim, and the Falcon tube geometry, along with the corresponding computational domain, was created. The computational domain was then divided into two zones with shared topology to ensure a conformal mesh. This step was necessary to define the rotor motion using a moving reference frame/mesh, representing the blade’s spinning in the CFD solver. A mesh study was performed, and approximately 2 million polyhedral cells were selected for both simulations to ensure that all residuals were below the convergence criterion, set at 0.00001. The computational model of RP motions was implemented into the finite volume method software, Ansys Fluent 2023 R1 (ANSYS, Canonsburg, PA).The incompressible Navier-Stokes equation serves as the governing equation to describe the flow field in the RPMotions. To account for rotational effects, as explained earlier, the multiple reference frame technique was applied with a rotational velocity of 60 rad/s. The coupled implicit algorithm was utilized to solve the momentum and pressure-based continuity equations simultaneously. The momentum equation and temporal term were discretized using the second-order upwind scheme and fully implicit second-order Euler scheme, respectively. The standard k-ε model was used for capturing turbulence effects. In line with the previous studies on computing phase fractions within simplex atomizers,^47^^,^^48^ the volume of fluid method (VOF) was used to numerically calculate the interface between the medium and air. By using a viscometer, the viscosity of the medium was determined to be 0.0013 Pa and it exhibited Newtonian fluid within a range of 0.1–1000 1/s. Therefore, both the medium and air were considered as Newtonian fluids in this study. The surface tension of water was incorporated as a source term in VOF equation to ensure continuous force at the interface. The implicit scheme was used to discretize the VOF equation.

#### Quantification of cell numbers

Manual cell counting was conducted for ICO and HSIO. In brief, 1 well or 500 μL of cell suspension was collected from static culture or RPMotion, respectively. After centrifugation and removal of the supernatant, 1 mL of TrypLE Express Enzyme was used to resuspend organoids and trypsinize organoids into single cells in a water bath at 37°C for 20–40 min. Once the organoids were almost completely trypsinized into single cells, 10 mL of cold basal medium was added to dilute TrypLE Express Enzyme and 0.25 mL FBS was added to each tube to protect single cells and help them to recover. After that, samples were centrifuged at a speed of 400 g for 5 min at 4°C. After removing the supernatant, single cells were resuspended by 200–500 μL and 30 μL cell suspension was used for cell counting with a TC20 automated cell counter (Bio-Rad) and counting slides. Similar to the preparation of single cells for ICO and HSIO, single cells of PDOs were counted with the same slides by a CytoSMART Exact platform following the manufacturer’s guidelines.

#### RNA isolation and quantitative real-time PCR (qPCR)

The RNeasy Mini Kit (Qiagen, Hilden, Germany) was used to isolate RNA from tissues and organoids following the manufacturer’s instructions. RNA quality and quantity were measured with DS-11 Spectrophotometer (DeNovix). Complementary DNA (cDNA) was synthesized with the iScript cDNA synthesis kit (Bio-Rad) following the manufacturer’s instructions. qPCR assays were conducted to determine the relative expression of target genes using validated primers using the SYBR Green method (Bio-Rad). Normalization was carried out using reference genes glyceraldehyde-3-phosphate dehydrogenase (*GAPDH*) and ribosomal protein L19 (*RPL19*).

Primers used for gene-expression profiling are listed in the table below.

**Table:** List of primers used for gene expression profiling

Target	Forward primer	Reverse primer	Annealing temperature, °C	Product size, bp
GAPDH	CAAGATCATCAGCAATGCCT	CAGGGATGATGTTCTGGAGAG	60	194
RPL19	ATGAGTATGCTCAGGCTTCAG	GATCAGCCCATCTTTGATGAG	64	150
LGR5	GCAGTGTTCACCTTCCC	GGTCCACACTCCAATTCTG	64	82
Ki67	GCTACTCCAAAGAAGCCTGTG	AAGTTGTTGAGCACTCTGTAGG	60	143
ECAD	AGGCCAAGCAGCAGTACATT	ATTCACATCCAGCACATCCA	60	110
HNF4a	CATGTACTCCTGCAGATTTAGCC	CTTCCTTCTTCATGCCAGCC	60	110
KRT19	CTTCCGAACCAAGTTTGAGAC	AGCGTACTGATTTCCTCCTC	64	183
HIF1a	CTGCCACCACTGATGAATTA	GTATGTGGGTAGGAGATGGA	57.2	90
SLC10A1	GATATCACTGGTGGTTCTC	ATCATCCTCCCTTGATGAC	60	100
ALB	GTTCGTTACACCAAGAAAGTACC	GACCACGGATAGATAGTCTTCTG	64	144
CYP3A4	CACAGGCTGTTGACCATCAT	TTTTGTCCTATAAGGGCTTT	60	92
MRP2	GCCAACTTGTGGCTGTGATAGG	ATCCAGGACTGCTGTGGGACAT	60	139
MDR1	AATGATGCTGCTCAAGTTAAAGGG	TCAGTAGCGATCTTCCCAGAACC	60	239
BSEP	TTGAGACAATAGACAGGAAACC	TCTGGAAGGATAATGGAAGGT	60	116
AXIN2	TGGATACAGGTCCTTCAAGAG	CGCATCACTGGATATCTCAC	60	112
VIL-1	ACTGTACCATGTGTCTGACTC	ATTGGCTTTCTTCCCTTTCC	64	150
OLFM4	TTCTTACACTGAACTGGACTTCG	ATATTTCTTATCTCCACCTCCAGC	64	134
MUC2	TGATGTCTGCGTGAAGACCT	CAGATGATGCCACTTCCACC	64	122
PDX1	CAGCTGCCTTTCCCATGGAT	TCCGCTTGTTCTCCTCCG	60	100
SOX9	CAAGCTCTGGAGACTTCTGAACG	CCGTTCTTCACCGACTTCCT	60	135
INS	GCAGCCTTTGTCAACCAACA	TTCCCCGCACACTAGGTAGAGA	60	69

#### Rhodamine123 transport assay

ICOs were differentiated for 8 days as previously described.^18^ For rhodamine123 (Rh123) transport assays, ICOs were pretreated with ICOD containing verapamil (10 μM, Sigma-Aldrich) or DMSO for 30 min. Organoids were then removed from Matrigel and resuspended in ICOD containing Rh123 (100 μM, Sigma-Aldrich) and incubated at 37°C for 10 min. Fluorescence was visualized by an EVOS FL Cell Imaging System (Life Technologies).

#### Ammonium elimination assay

For ammonium elimination assays, ICOs were differentiated in Matrigel droplets for 8 days as previously described.^18^ ICOs were incubated with ICOD supplemented with NH_4_Cl (2 mM) for 24 h. After 24 h, medium samples were harvested and stored at −20°C. Afterward, Tryple-Express (Gibco) was added to each well, and ICOs were trypsinized for cell counting. Cell counts were carried out using the TC20 automated cell counter (Bio-Rad). Viable cells were determined using a trypan blue exclusion assay. Ammonium concentrations were measured with the Urea/Ammonia Assay Kit (Megazyme). As a control, ICOD containing NH_4_Cl (2 mM) was incubated for 24 h without cells. Ammonia elimination rates were normalized to live cell numbers.

#### GLDH expression and albumin production

To quantify the intracellular levels of GLDH and albumin, HLOs were differentiated in Matrigel droplets for 8 days, as previously described.^18^ Organoids were provided with fresh DM 24 h before being lysed in MilliQ water. GLDH and albumin (ALB) were measured in the cell lysates using a DxC-600 Beckman chemistry analyzer (Beckman Coulter). Values were normalized to total protein concentrations.

#### Microscopy and immunofluorescence (IF) analysis

Imaging of the organoids was performed using an EVOS FL Cell Imaging System (Life Technologies), and an Olympus BX51 microscope in combination with an Olympus DP73 camera. Detailed information on applied antigen retrieval methods, antibodies, dilutions, and incubation times are listed in Table.

Bright-field images were taken to track organoid morphology throughout expansion and differentiation for different types of organoids in both static cultures and RPMotion. Images were also taken to compare the morphology of organoids in EM and DM.

For IF staining, organoids were fixed with 4% (v/v) neutral buffered formalin containing 0.1% eosin at room temperature (RT) for 1 h. Fixed samples were dehydrated and embedded in paraffin or stored in 70% (v/v) ethanol at 4°C for up to 1 month; 4–5 μm thick paraffin sections were prepared for IF staining. To start the IF staining procedure, the paraffin sections were first heated at 62°C for 10 min and dewaxed by xylene, followed by rehydration in gradient ethanol concentrations from 100% to 70%, and lastly in MilliQ water for 5 min. Then, sample sections were incubated in antigen retrieval solution for 30 min at 98°C. After balancing to room temperature, sample sections were treated with NH_4_Cl solution (20 mM) for 20 min to reduce background autofluorescence and blocked with 10% (v/v) goat serum for 1 h to avoid non-specific antibody binding. Next, primary antibodies against E-cadherin (ECAD), Ki67, PCNA, KRT19 (CK19), ALB, MRP2, LYZ, VIL-1, etc. were added to the sections and incubated overnight at 4°C. After being washed with PBS containing 0.1% (v/v) Tween 20 (PBST) three times for a total of 15–20 min, sample sections were incubated with secondary antibodies (5 μM), including mouse anti-rabbit Alexa Fluor488 (Molecular Probes), mouse anti-rabbit Alexa Fluor647 (Molecular Probes), rabbit anti-mouse Alexa Fluor488 (Molecular Probes) and rabbit anti-mouse Alexa Fluor647 (Molecular Probes). Nuclei were stained with DAPI (0.5 μg/mL, Sigma Aldrich).

Antibodies used are listed in the table below.

**Table:** List of antibodies used

Primary antibodies diluted in antibody diluent (Dako) for IF					
Antigen	Source and catalog no.	Raised in	Dilution	Antigen retrieval	Incubation
Ki67	Thermo Scientific, RM-9106-S	rabbit	1:75	Tris-EDTA buffer (pH 9) for 30 min at 98°C	overnight at 4°C
E-cadherin	BD Bioscience, 610181	mouse	1:100		
PCNA	Santa Cruz, sc-56	mouse	1:300		
K19	Abcam, ab76539	rabbit	1:150		
ALB	Sigma, A6684	mouse	1:1,000		
MRP2	Abcam, ab187644	rabbit	1:1,000		
LYZ	Abcam, ab74666	rabbit	1:200		
VIL-1	Abcam, ab201989	mouse	1:500		
SOX9	LSbioscciences, LS-C148618	rabbit	1:250		

Secondary antibodies diluted in antibody diluent (Dako)				
Antigen	Source and catalog no.	Raised in	Dilution	Incubation
Anti-mouse Alexa 488	Thermo Fisher A-11029	goat	1:200	1 h at room temperature
Anti-rabbit Alexa 488	Thermo Fisher A-11034			
Anti-mouse Alexa 568	Thermo Fisher A-11004			
Anti-rabbit Alexa 568	Thermo Fisher A-11036			

### Quantification and statistical analysis

Cell quantification data were analyzed in an Excel datasheet and converted into graphs using GraphPad Prism 9. qPCR results, albumin secretion, ALT and AST levels, GLDH expression, and ammonium elimination were analyzed using two-way ANOVA multiple comparisons tests. The exact tests (Tukey’s multiple comparisons test or Šídák’s multiple comparisons test) are listed in the respective figure legends. Graphs indicate mean ± SD. The *p*-values are indicated in the respective figures.

## Supplemental information

Document S1. Figures S1–S8

Document S2. Article plus supplemental information
