# Supplementary material for: Accelerated production of human epithelial organoids in a miniaturized spinning bioreactor
Source: Cell Rep Methods. 2024 Nov 18;4(11):100903. doi: 10.1016/j.crmeth.2024.100903 (PMC11705766; doi:10.1016/j.crmeth.2024.100903)
Supplement: Document S1. Figures S1–S8 [file mmc1.pdf]

Cell Reports Methods, Volume *4*

Supplemental information

Accelerated production of human epithelial organoids in a miniaturized spinning bioreactor

Shicheng Ye, Ary Marsee, Gilles S. van Tienderen, Mohammad Rezaeimoghaddam, Hafsah Sheikh, Roos-Anne Samsom, Eelco J.P. de Koning, Sabine Fuchs, Monique M.A. Verstegen, Luc J.W. van der Laan, Frans van de Vosse, Jos Malda, Keita Ito, Bart Spee, and Kerstin Schneeberger

SUPPLEMENTAL INFORMATION

Supplemental information is now following the **STAR * METHODS** and will be provided in the online version of this manuscript.

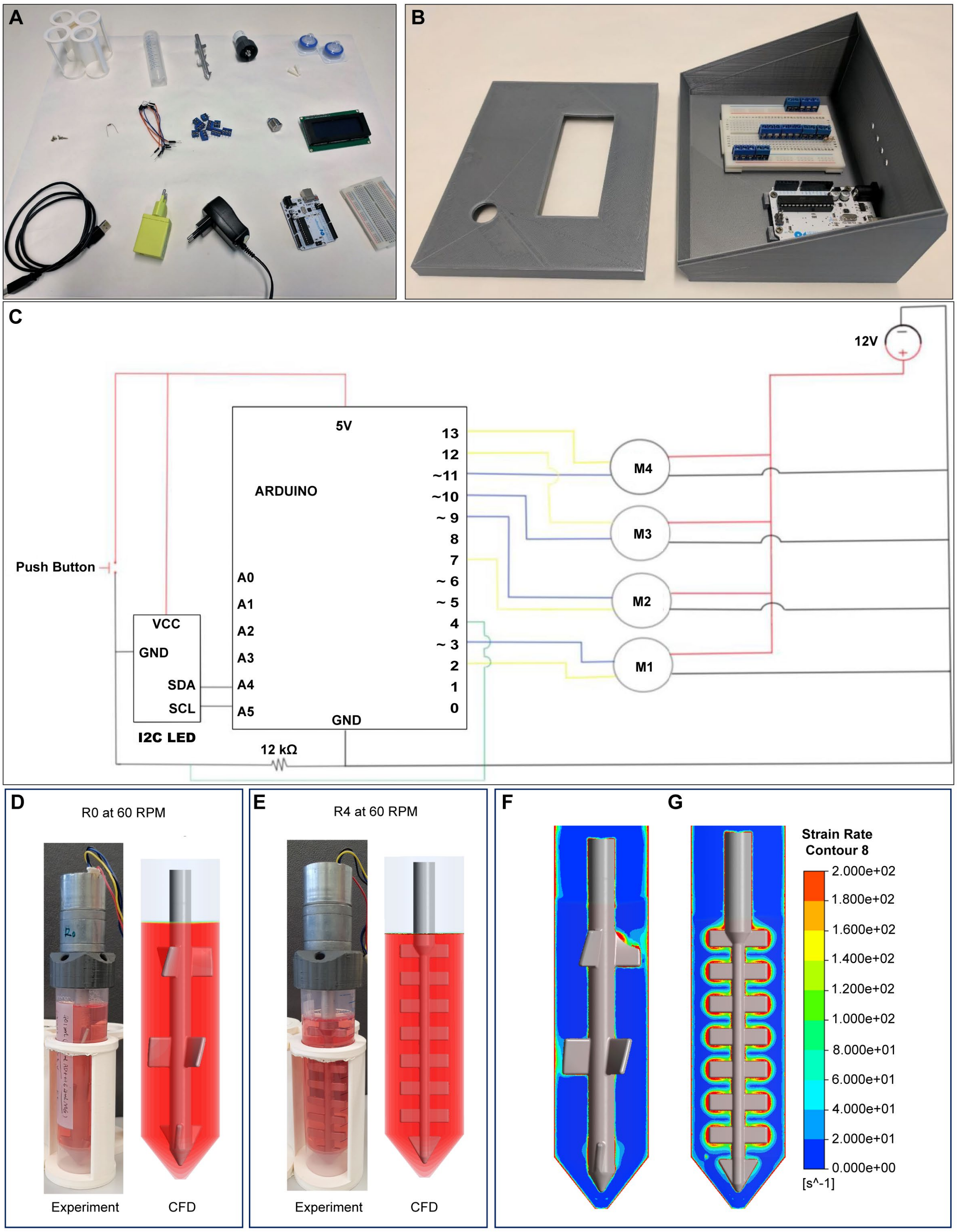

Figure S1. Assembly of the RPMotion and analysis of bioreactors with different rotors, related to Figure 1.

(A) Parts used to assemble the RPMotion (excluding enclosure and motor connections).

(B) RPMotion enclosure top (left) and bottom (right) containing Arduino Uno and breadboard (with screw terminals attached).

(C) Circuit schematic showing how the motors, pushbutton, LCD screen, and Arduino Uno are connected.
(D-E) Experimental and computational results for RPMotion bioreactors with R0 (D) and R4 (E) both at 60 rpm. Photos were taken at a sufficient time to consider a steady state condition as well as the visualization of phase fraction of medium-air computational results.
(F-G) Strain rate (*i.e.* fluid shear strain) contour comparison of rotor R0 (F) to R4 (G).

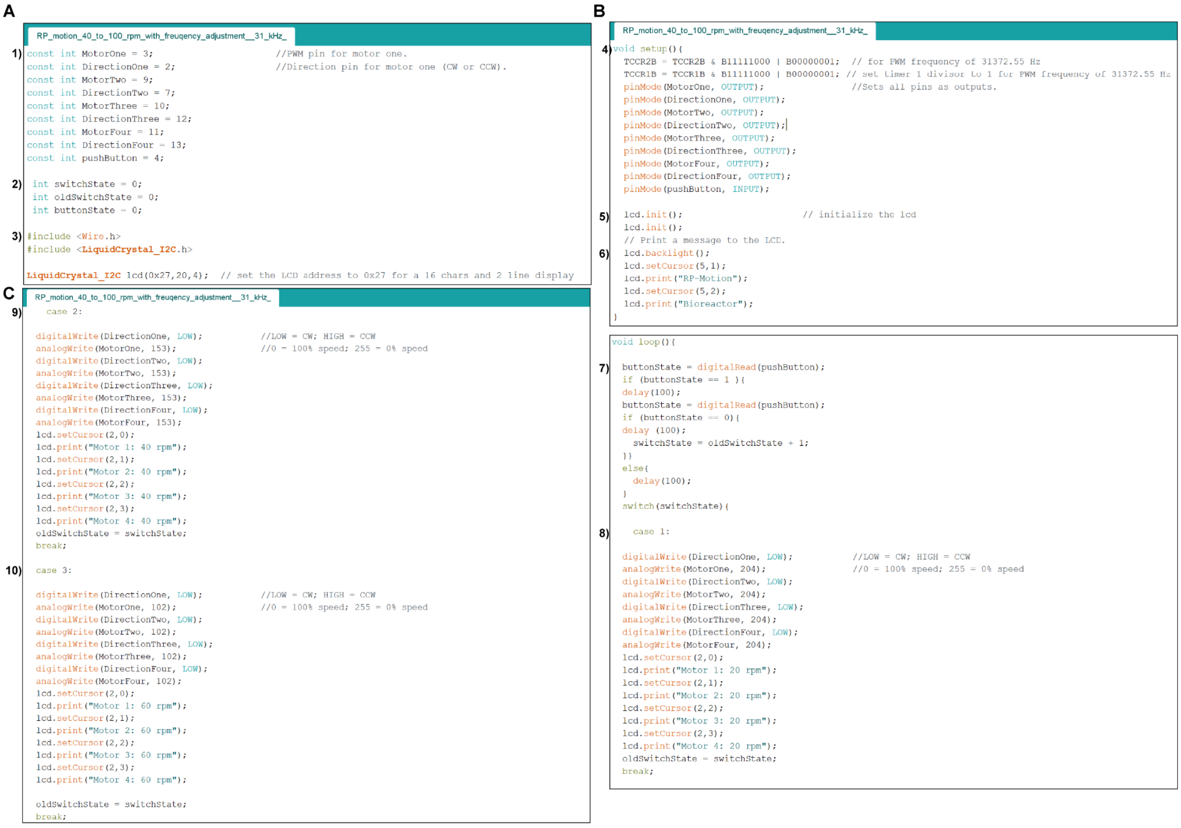

Figure S2. Software and a sample program to run the RPMotion bioreactors, related to Figure 1. (A) 1) Defines the pins for the direction and PWM signal of each motor as well as the push-button pin. 2) Defines the pushbutton states. 3) Includes the libraries for I2C control of the LCD screen. (B) 4) Sets the frequency of the PWM pins to 31,372 Hz. 5) Sets the motor pins as outputs and the pushbutton pin as an input. 6) Initializes the LCD screen and prints “RP-Motion Bioreactor” on the screen. 7) Reads the state of the pushbutton and switches the state if the button has been pressed. 8) Case 1: sets each motor to rotate at 40 rpm in the CW direction. Also displays the name of each motor along with speed on the LCD. (C) 9) Case 2: sets each motor to rotate at 60 rpm in the CW direction. Also displays the name of each motor along with speed on the LCD. 10) Case 3: sets each motor to rotate at 80 rpm in the CW direction. Also displays the name of each motor along with speed on the LCD. Cases 4 and 5 are not displayed.

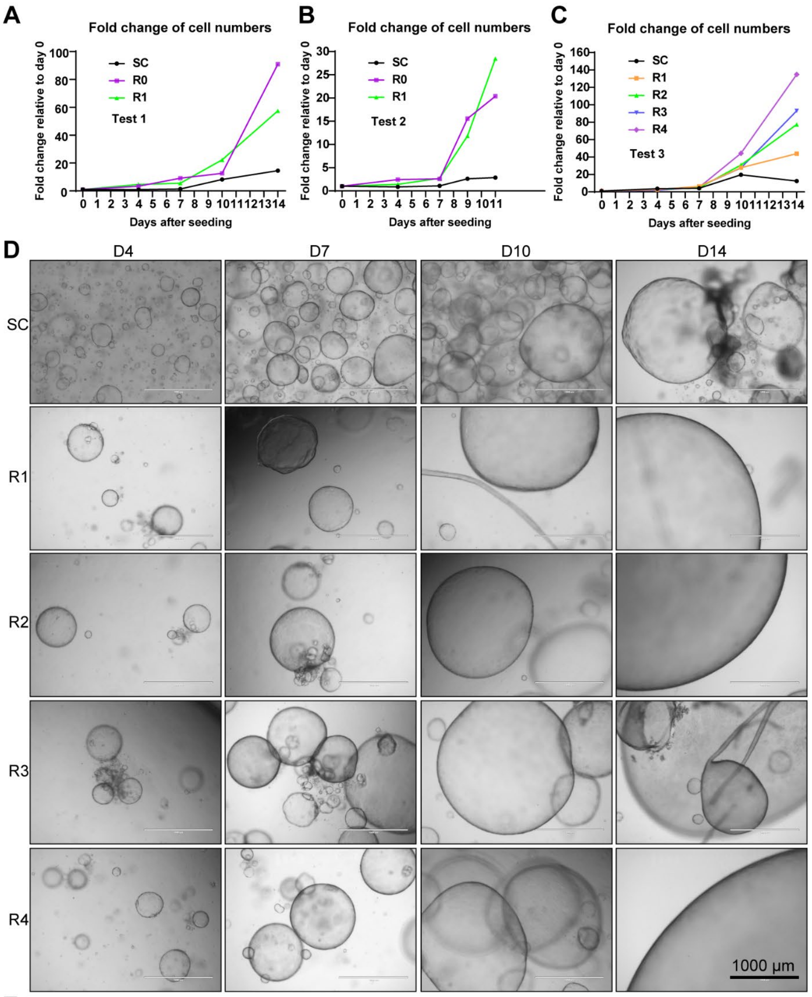

E

Test 1	D0	D1	D2	D3	D4	D5	D6	D7	D8	D9	D10	D11	D12	D13	D14
SC*10^5 cells/well	0,25				0,22			0,27		0,65		0,71			
R0*10^5 cells/ml	0,50				0,61			0,53		1,62		1,42			
R1*10^5 cells/ml	0,50				0,36			0,56		1,23		1,98			

Test 2	D0	D1	D2	D3	D4	D5	D6	D7	D8	D9	D10	D11	D12	D13	D14
SC*10^5 cells/well	0,25				0,25			0,35			2,05				3,65
R0*10^5 cells/ml	0,50				1,05			1,82			1,50				7,58
R1*10^5 cells/ml	0,50				1,46			1,10			2,65				4,79

Test 3	D0	D1	D2	D3	D4	D5	D6	D7	D8	D9	D10	D11	D12	D13	D14
SC*10^5 cells/well	0,25				0,91			1,06			4,91				3,11
R1*10^5 cells/ml	0,50				0,86			1,26			3,16				3,43
R2*10^5 cells/ml	0,50				0,93			0,95			3,50				6,04
R3*10^5 cells/ml	0,50				0,62			1,21			3,12				7,28
R4*10^5 cells/ml	0,50				0,62			1,06			5,03				10,54

Figure S3. Selection of the optimal rotor (R) for the RPMotion bioreactor, related to Figure 2.

(A) Comparison of the original tilted R0 and updated flat R1 with static culture for the expansion of human liver organoids (ICOs, donor 2) in the bioreactor at 60 rpm. Proliferative curves are shown compared to day 0 (D=day. D0) after single-cell seeding.

(B) A second donor (donor 4) was tested for the comparison as in (A).

(C) Comparison of different rotor designs (R1, R2, R3, R4) to static culture for the expansion of human liver organoids (ICOs, donor 4) in the bioreactor at 60 rpm. Proliferative curves based on cell counting at different time points.

(D) Morphological pictures of ICOs (donor 4) expanded in the bioreactor with four different rotors at 60 rpm at four different time points (D4, D7, D10, D14). Scale bar, 1000 μm.

(E) Original cell counts of tests 1, 2, and 3 shown in (A), (B), (C).

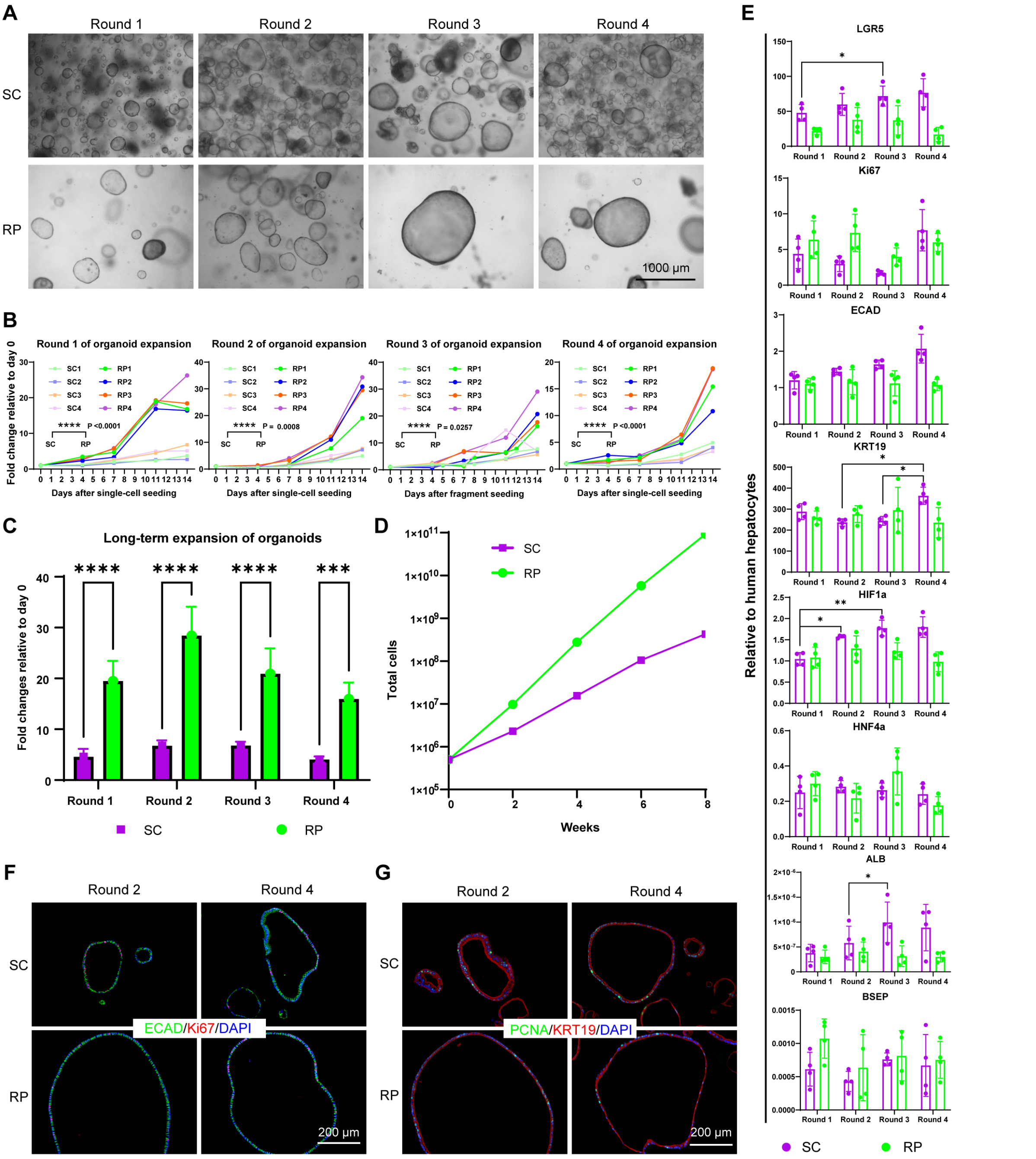

Figure S4. Long-term expansion of ICOs in the RPMotion bioreactor, related to Figure 2.

(A) Morphological pictures of ICOs expanded in static culture and in the bioreactor for 4 rounds (R1, R2, R3, R4), with passaging after each round of expansion. As it takes 2 weeks per round, the long-term expansion lasted 8 weeks at 60rpm. Bright field photos shown were taken at the end of each round (D14) after single cell seeding. Scale bar, 1000 μm.

(B) Proliferative curves are shown as fold changes of cell numbers compared to D0 of each round. Cell counts were analyzed at four time points (D4, D7, D11, D14) during each round of expansion. Four donors were used (with numbers 1, 2, 3, and 4). 2way ANOVA analysis was applied.

(C) Average fold changes of ICO cell numbers in the static culture and in the bioreactors per round of expansion during 8 weeks. 2way ANOVA Šídák's multiple comparisons test was applied for the analysis. Graphs indicate mean ± SD. ***p = 0.0001, ****p < 0.0001.

(D) The theoretical accumulated cell mass during 8 weeks of ICO expansion.

(E) Gene expression characterized by qPCR assays. Tukey's multiple comparisons test was applied. Graphs indicate mean ± SD. *$p < 0.05$, **$p < 0.001$.

(F-G) Immunofluorescent staining (IF) pictures of ICOs after 4 weeks (R2) and 8 weeks (R4) of expansion in the static culture or in the bioreactors. Displayed are ductal (KRT19), epithelial (ECAD), and proliferative (Ki67 and PCNA) markers. n=4. Scale bar, 200 μm.

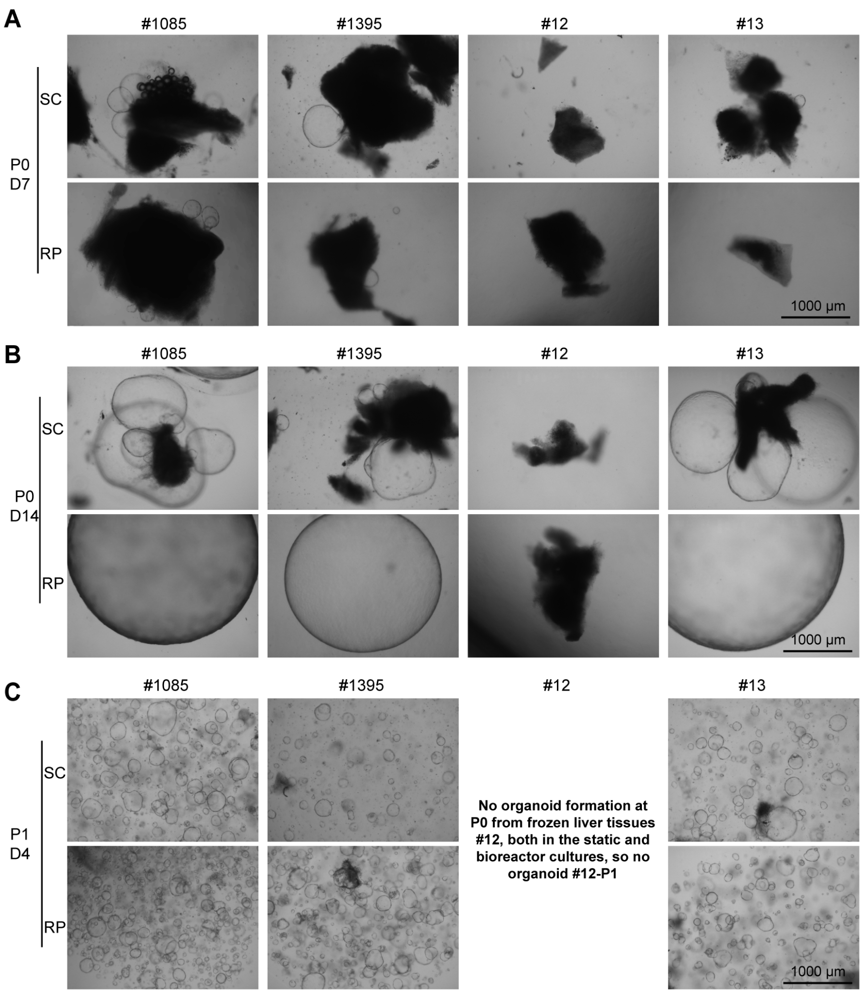

Figure S5. New ICO lines can be established directly in the bioreactor, related to Figure 2.
(A-B) Morphological pictures of new ICO lines (passage 0, P0) established from human liver tissues in static cultures or in the bioreactors. Human liver tissue #1085 was fresh, and #1395, #12 and #13 were thawed from frozen biopsies. Brightfield photos were taken at D7 (A) and D14 (B).
(C) Morphological pictures of ICOs at day 4 after passaging and re-seeding in Matrigel droplets. Scale bar, 1000 μm.

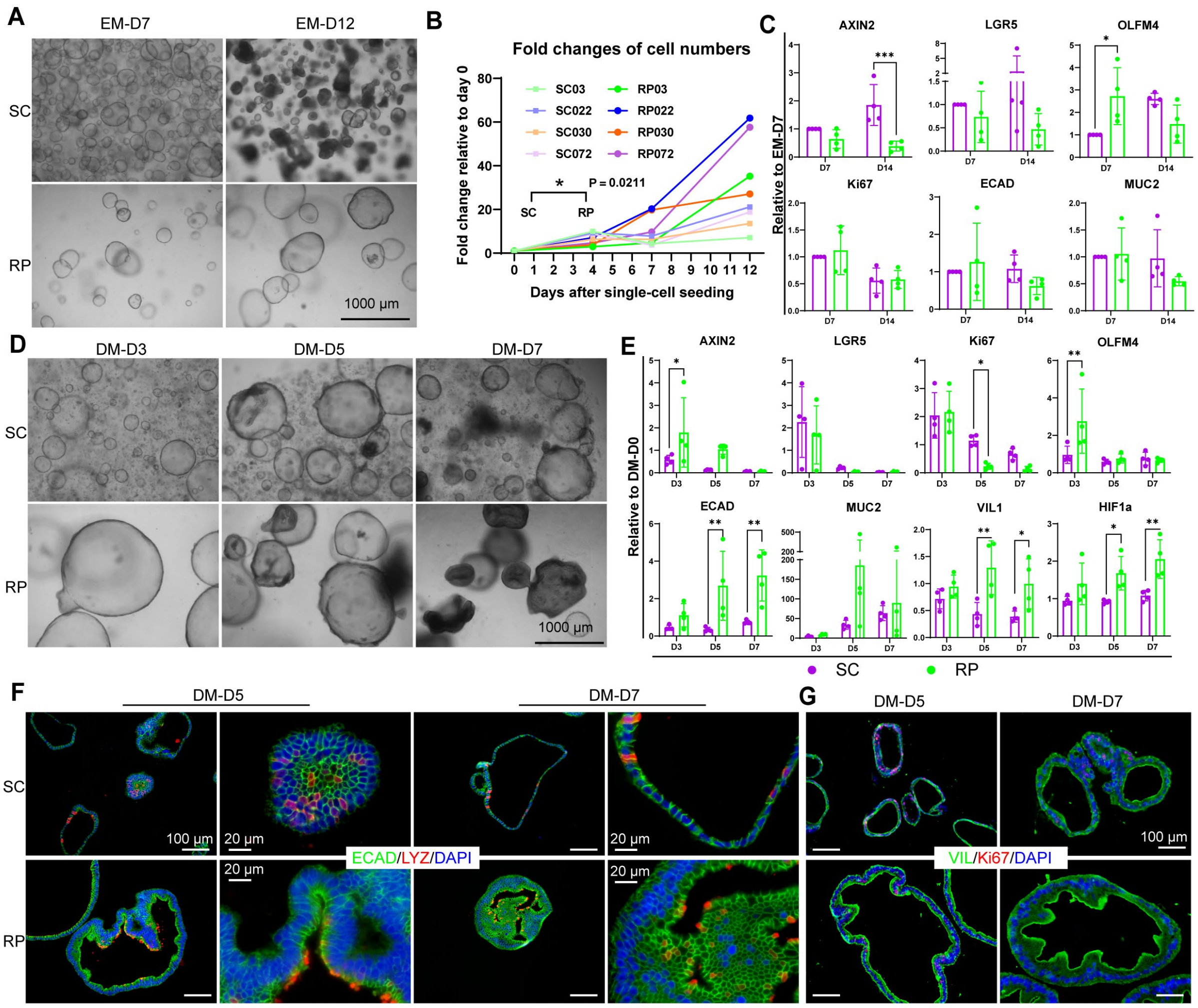

Figure S6. The bioreactor is efficient for HSIO expansion and differentiation, related to Figure 2 and 3.

(A) Morphological pictures of human small intestinal organoids (HSIOs) expanded in the RPMotion bioreactor (100 rpm) or static culture at two time points (D7 and D12). Scale bar, 1000 μm.

(B) Proliferative curves were made based on cell counting at three time points (D4, D7, D12). 2way ANOVA analysis was applied.

(C) Gene expression of HSIOs after 7 (EM-D7) or 14 (EM-D14) days of expansion, characterized by qPCR assays. 2way ANOVA Šídák's multiple comparisons test was applied for the analysis. Graphs indicate mean ± SD. **p* < 0.05, ****p* = 0.0006.

(D) Morphological pictures of HSIOs after 5 (DM-D5) or 7 (DM-D7) days of differentiation in the static culture or in the bioreactor. Scale bar, 1000 μm.

(E) qPCR results of HSIO gene expression after differentiation for 3 (D3), 5 (D5), and 7 (D7) days. 2way ANOVA Šídák's multiple comparisons test was applied for the analysis. Graphs indicate mean ± SD. **p* < 0.05, ***p* < 0.01.

(F-G) Immunofluorescent staining (IF) pictures of HSIOs after differentiation for 5-7 days. Epithelial protein ECAD and Paneth cell protein lysozyme (LYZ) are shown in F, and microvillus marker villin 1 (VIL1) and proliferative marker Ki67 are shown in G. n=4. Scale bar, 100 μm if not further specified; or 20 μm where indicated in the pictures.

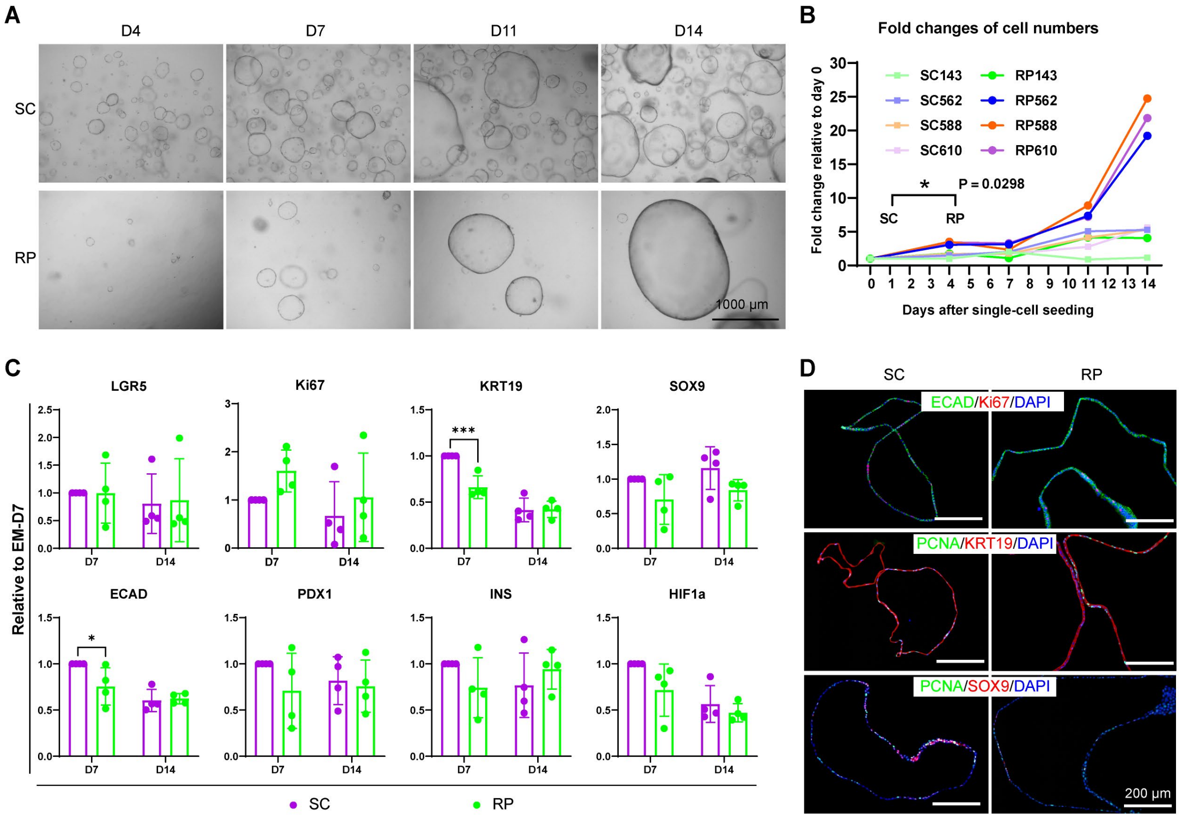

Figure S7. The bioreactor is efficient for PDO expansion, related to Figure 1.

(A) Morphological pictures of pancreatic ductal organoids (PDOs) expanded in the RPMotion bioreactor (80 rpm) or static culture at 4 different time points (D4, D7, D11, D14). Scale bar, 1000 µm.

(B) Proliferative curves were made based on cell counting at different time points. 2way ANOVA analysis was applied.

(C) Gene expression levels characterized by qPCR assays. 2way ANOVA Šídák's multiple comparisons test was applied for the analysis. Graphs indicate mean ± SD. *$p < 0.05$, ***$p < 0.001$.

(D) Immunofluorescent (IF) staining pictures of PDOs after 14 days of expansion in the static culture or in the bioreactors. n=4. Scale bar, 200 µm.

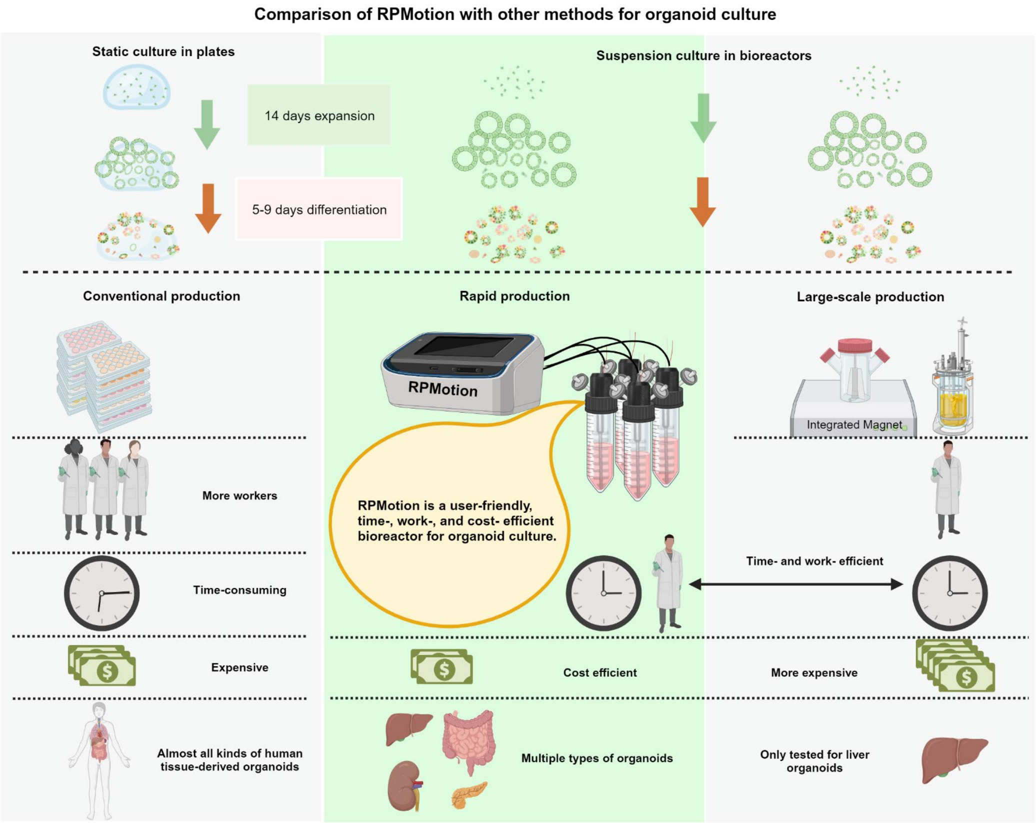

Figure S8. An overview of comparing the RPMotion bioreactor with other methods for human organoid culture, related to STAR METHODS.
